# WGNAM: whole-genome nested association mapping

**DOI:** 10.1007/s00122-022-04107-x

**Published:** 2022-05-21

**Authors:** M. Valeria Paccapelo, Alison M. Kelly, Jack T. Christopher, Arūnas P. Verbyla

**Affiliations:** 1 Department of Agriculture and Fisheries, Leslie Research Facility, Toowoomba, QLD 4350 Australia; 2 Queensland Alliance for Agriculture and Food Innovation (QAAFI), The University of Queensland, Leslie Research Facility, Toowoomba, QLD 4350 Australia; 3 AV Data Analytics, Pilton, QLD 4361 Australia; 4 Queensland Alliance for Agriculture and Food Innovation (QAAFI), The University of Queensland, St. Lucia, Brisbane, QLD 4067 Australia

## Abstract

**Key message:**

A powerful QTL analysis method for nested association mapping populations is presented. Based on a one-stage multi-locus model, it provides accurate predictions of founder specific QTL effects.

**Abstract:**

Nested association mapping (NAM) populations have been created to enable the identification of quantitative trait loci (QTL) in different genetic backgrounds. A whole-genome nested association mapping (WGNAM) method is presented to perform QTL analysis in NAM populations. The WGNAM method is an adaptation of the multi-parent whole genome average interval mapping approach where the crossing design is incorporated through the probability of inheriting founder alleles for every marker across the genome. Based on a linear mixed model, this method provides a one-stage analysis of raw phenotypic data, molecular markers, and crossing design. It simultaneously scans the whole-genome through an iterative process leading to a model with all the identified QTL while keeping the false positive rate low. The WGNAM approach was assessed through a simulation study, confirming to be a powerful and accurate method for QTL analysis for a NAM population. This novel method can also accommodate a multi-reference NAM (MR-NAM) population where donor parents are crossed with multiple reference parents to increase genetic diversity. Therefore, a demonstration is presented using a MR-NAM population for wheat (*Triticum aestivum* L.) to perform a QTL analysis for plant height. The strength and size of the putative QTL were summarized enhancing the understanding of the QTL effects depending on the parental origin. Compared to other methods, the proposed methodology based on a one-stage analysis provides greater power to detect QTL and increased accuracy in the estimation of their effects. The WGNAM method establishes the basis for accurate QTL mapping studies for NAM and MR-NAM populations.

**Supplementary Information:**

The online version contains supplementary material available at 10.1007/s00122-022-04107-x.

## Introduction

The identification of quantitative trait loci (QTL) has been an important step toward understanding the underlying genetics of traits of agronomic interest for crops and has become an important breeding tool to release improved lines. For decades, QTL detection has been successfully conducted by traditional QTL mapping in bi-parental populations known as linkage analysis (Takeda and Matsuoka 2008; Collard et al. 2005; Bernardo 2002). However, the detected chromosome regions were generally broad and not always transferable to other genetic backgrounds (Yu et al. 2008). Alternatively, genome-wide association studies (GWASs) have been performed to identify QTL using diversity panels, based on a genetically diverse pool of individuals with unknown kinship. The use of diversity panels explores broader genetic backgrounds and benefits from historic recombination events, potentially providing higher mapping resolution. Nonetheless, it can fail to capture alleles of interest that are present at low frequencies and the unknown population structure can compromise the associations between marker and trait. More recently, the strengths of both QTL mapping approaches have been integrated through the development of multiple connected populations (Blanc et al. 2006), also known as multi-parent (MP) populations. This strategy aims to capture more recombination events and greater allelic diversity than a single bi-parental population. Additionally, compared to a diversity panel, some rare alleles are brought to moderate frequencies and the population structure is known from the crossing design.

Different types of MP populations have been developed in crops following different crossing designs. Blanc et al. (2006) proposed a half-diallel design where all parents were crossed with each other and reciprocals were not considered. Nested association mapping (NAM) populations were first discussed in maize by Yu and Buckler (2006). The development of a NAM population starts by selecting a set of diverse donor parents (donors) to be crossed to a reference elite line (reference) in order to obtain multiple families of recombinant inbred lines (RILs) that are related by the common reference parent. Another example is the multi-parent advanced generation integrated cross (MAGIC) population first described in crops by Cavanagh et al. (2008). A classic MAGIC population design starts with a selection of diverse founder lines that are then inter-crossed until all founders have an equal probability of contributing to the genetic makeup of a line and this is followed by multiple generations of self-pollination to create RILs. Other mating designs have been used to perform QTL analysis in crops, which do not follow any particular pattern (Bardol et al. 2013), while others are variations of previous designs (Jordan et al. 2011; Mace et al. 2013). An example of a variation is the multi-reference nested association mapping (MR-NAM) population which has been developed in wheat consisting of several related NAM populations. Several different reference parents were used while some donors were shared across NAM populations to keep them inter-connected (Richard 2017). The resulting MR-NAM population enables the exploration of alleles from multiple donors and several genetic backgrounds.

The simplest analysis of an MP population treats the population as a diversity panel using the GWAS method. In general, GWAS methods scan the whole genome by testing for association between the phenotypic data and every molecular marker. Despite being easy to implement, GWAS has several issues. Firstly, the increased false-positive rate due to multiple testing demands some correction to control it; two commonly used methods are the Bonferroni correction (Holm 1979) and false discovery rate correction (Benjamini and Hochberg 1995). Secondly, a single-locus approach fails to explain complex traits that are controlled by numerous loci simultaneously, and as such multi-locus models are preferred (Segura et al. 2012; Wang et al. 2016; George et al. 2020). Finally, failure to account for the crossing design results in false positives due to the population structure and family relatedness (Blanc et al. 2006). Hence, the population structure needs to be inferred from the molecular markers and incorporated into the model using one of several suggested approaches (Pritchard et al. 2000; Price et al. 2006; Yu and Buckler 2006). Even when these issues are addressed, a major limitation of GWAS methods in MP populations is that they generally use bi-allelic marker models which can fail to reflect the population allele diversity (Garin et al. 2017).

For the analysis of an MP population, it is crucial to determine how best to incorporate the phenotypic data, the crossing design, and the generally large number of molecular markers in an appropriate statistical model. Various statistical models have been developed to perform QTL analysis for MP populations. In NAM populations, for instance, Yu et al. (2008) simply incorporated a covariate accounting for family effects. Buckler et al. (2009) introduced the estimation of marker effects nested within each family in the NAM population and proposed a method called joint inclusive composite interval mapping that used significant markers as co-factors to account for the background genetic variance. Xavier et al. (2015) proposed performing a GWAS with molecular markers recoded to consider the number of alleles coming from each founder of the NAM population. Additional methods were developed for other crossing designs and these allow for different assumptions around the allelic effect of the founders. Some examples of the assumptions behind different methods are, (i) assuming that each founder has the same allelic effect independently of the background or family (Jourjon et al. 2005), (ii) accepting founders can be related and using ancestral alleles as implemented in the software package MCQTL combined with the R-package Clusthaplo (Leroux et al. 2014), and (iii) a mixture of different assumptions as implemented in the MPP (multi-parent population) R-package (Garin et al. 2018). More recently, Li et al. (2021) presented an identity-by-decent-based mixed model approach that is flexible enough to be used for all kinds of MP populations and this method was competitive with other tools developed for specific MP populations. While each of these methods has the advantage of accounting for the crossing design based on the assumptions of the allelic effects, they still need to define a correction to control the false positive rate. The major disadvantage however is that they all follow a two-stage approach.

QTL mapping methods can follow a one-stage or two-stage approach. In a two-stage approach, the analysis of experimental phenotypic data is performed first (Stage 1). The genotype means are then used as the response variable in Stage 2, where associations with molecular markers are tested. Typically the variance-covariance of the genotype means predicted in Stage 1 are not incorporated into the second-stage model. This compromises the full analysis, particularly in the presence of sophisticated models for the genetic effects (Gogel et al. 2018). A one-stage analysis that models the entire observed data at the level of individual plots is usually considered as the gold standard (Piepho et al. 2012). Furthermore, Gogel et al. (2018) concluded that a one-stage analysis is crucial for trials with minimal replication such as those with partially replicated treatments. Partially replicated designs have become the experimental design of choice in many crops (Mace et al. 2013; Lehermeier et al. 2014; Verbyla et al. 2014b; Richard 2017) because the size of an MP population can reach the order of thousands of lines, each needing to be tested in the same experimental trial.

To our knowledge, the only one-stage approach implemented in MP populations is the multi-parent whole-genome average interval mapping (MPWGAIM) method developed for MAGIC populations and implemented in the MPWGAIM R-package (Verbyla et al. 2014b). As an extension of the whole-genome average interval mapping method for bi-parental populations, MPWGAIM is based on a linear mixed model. This enables the ability to simultaneously incorporate non-genetic sources of variation such as experimental design effects or multi-phase data generation processes (Verbyla et al. 2007, 2012). The MPWGAIM method has been demonstrated to be a powerful tool for QTL mapping in MAGIC populations as it utilizes the probability of inheriting founder alleles across the whole genome simultaneously, either for intervals or markers.

In this paper, a whole-genome nested association mapping (WGNAM) analysis method is presented as an adaptation of the MPWGAIM approach, to perform association studies for NAM populations. This method, based on a linear mixed model, provides a one-stage analysis of raw phenotypic data, molecular markers, and population design. It simultaneously scans the whole-genome through an iterative process, leading to a multi-locus model using the full set of putative QTL, without the need to perform multiple testing corrections. It is versatile enough that it can easily accommodate the MR-NAM population structure and has the potential to accommodate other derived crossing designs. The WGNAM approach was evaluated through a simulation study as well as real data. The simulation study was implemented to examine the false positive rate and the power to detect true QTL based on a NAM population structure. The performance of WGNAM was compared with a method that can deal with the NAM population structure, the MPP method (Garin et al. 2017), as well as with a GWAS approach, the multi-locus mixed model or MLMM (Segura et al. 2012). The WGNAM method was then illustrated using a real wheat MR-NAM population in order to map QTL for plant height. The WGNAM approach has the potential to significantly improve QTL mapping studies for NAM and MR-NAM populations because it increases the power to detect QTL, prevents the loss of information entailed in two-stage analysis, and acknowledges the complexity of the population structure while controlling the Type I error.

## Materials

The WGNAM methodology presented in this paper is examined utilizing empirical data consisting of the wheat multi-reference nested association mapping (MR-NAM) population first described by Richard (2017) and developed at the Queensland Alliance for Agriculture and Food Innovation (QAAFI). Additionally, a NAM population was simulated based on a subset of founders from the wheat MR-NAM to create realistic marker profiles.

### Plant material

The development of the wheat MR-NAM population is fully described by Richard (2017) and Christopher et al. (2021). Briefly, the parents of the MR-NAM population or founder lines (founders) were selected for attributes desirable for improving and expanding wheat production in diverse environments, such as those found in the Australian wheat belt (Table 2). Three reference parents, namely Suntop, Mace and Scout, were selected as key commercial lines in the Eastern, Southern, and Western Australian wheat production regions, respectively. Eleven donors were selected for traits including stay-green, favorable root architecture, disease resistance, and tolerance to drought and heat.

The MR-NAM population was developed by crossing the three reference parents with the 11 donors following an incomplete crossing scheme that produced a total of 20 F1 crosses. Dwarfing genes presence in the founders was tested following Ellis et al. (2002) (Table 2). Differences in the dwarfing genes present in the founders meant that segregation could result in double dwarf or tall genotypes rather than the preferred semi-dwarf phenotype. To provide an agronomically relevant phenotype for comparisons in subsequent yield testing in the field, a moderate selection pressure (around one plant in four selected) was applied in F2 bulks for semi-dwarf plant-height. Plants were also moderately selected for maturity to resemble the respective reference parent. The selected F2s were then progressed to the F4 generation by following the single seed descent method. The subsequent RIL populations served as families within the MR-NAM population with sizes varying from 33 up to 51 lines. Table 2 includes the founder lines and their dwarfing gene status for Rht-B1b (formerly Rht1) and Rht-D1b (formerly Rht2).

### Markers and map

Recurrent inbred lines were genotyped using the Diversity Arrays Technology (DArT) Pty Ltd (DArT 2017) wheat genome-by-sequencing platform. A single F4 plant representing each NAM line was sampled for leaf tissue, and DNA was isolated using the CTAB-based extraction protocol recommended by DArT. The genotype-by-sequencing process was carried out for a larger set of lines within which the MR-NAM population lines were a subset and it generated 18,827 single nucleotide polymorphism(s) (SNPs). A subset of markers was successfully positioned on the wheat DArT consensus map v.4 provided by Dr. Andrzej Killian (DArT 2018) consisting of 21 linkage groups (LGs).

A quality control and imputation process was performed on the marker data corresponding to the founders and MR-NAM population under analysis. SNPs were removed if they were not positioned on the consensus genetic map (approximately 30% of the initial 18,827 SNPs). SNPs were also excluded if they were not consistent among marker profiles of different samples of the same founder or presented a missing rate of either above 50% within family or over 10% overall (approximately 25% of the 18,827 SNPs were removed). Some SNPs were disregarded based on inconsistencies between SNP genotypes from family and parents (approximately 5%). For the founders, heterozygous SNP genotypes were considered missing data, and then missing SNP genotypes were imputed following a random forest process implemented in the NAM package (Xavier et al. 2015) in R (R Core Team 2019). SNP genotypes for the MR-NAM lines, were imputed based on the parental information first and when uncertain, via the random forest process. Finally, SNPs with a minor allele frequency of less than 0.01 across families, including fixed alleles, were excluded from the analysis (approximately 15%). Hence, approximately 20% of the initial 18,827 SNPs were used in the association studies.

### MR-NAM founder probabilities calculation

A way to formulate a QTL analysis that accounts for the crossing design is to define QTL effects in terms of their origins. Verbyla et al. (2014b) proposed to use the probabilities of inheriting founder alleles also known as identical by descent (IBD) probabilities for each potential QTL locus and each line in a MAGIC population. In this paper, the approach to estimate the IBD probabilities requires marker genotypes for the founders and every line within the family. It is assumed that founders are inbred lines and families were developed following the NAM crossing scheme (Yu et al. 2008) but no specification is required regarding the number of self-pollination cycles. This approach treats each marker independently from the others hence, no genetic map or physical map are needed. However, there are methods that use these maps to estimate the probabilities (Broman et al. 2003; Verbyla et al. 2014b; Li et al. 2021).

Given a family, if both founders have different genotype at a given position then, lines with homozygous genotypes will have inherited the alleles from just one of the founders with a probability equal to 1 and 0 for the other one (see Table 1, Cross Founder 1 $\times$ Founder $\mathrm {n_f}$, lines 1 and 2 as an example), whereas lines with heterozygous genotypes will have probability equal to 0.5 for each of the two founders of that cross (Table 1, Cross Founder 1 $\times$ Founder $\mathrm {n_f}$ line $\mathrm n_1$). If both founders of a family have the same genotype at a given position, then all the lines in that cross will have the same genotype and the method will assign the same probability of having an allele coming from either of the two parents (Table 1, Cross Founder 2 $\times$ Founder $\mathrm {n_f}$).

**Table 1:** Examples of the process used to calculate the probabilities of inheriting founder alleles in NAM populations at a given marker

Example	Cross (Donor $\times$ Reference)	Line	Marker genotype			Founder probabilities			
			Donor	Reference	Line	Founder 1	Founder 2	.	Founder $\mathrm {n_f}$
A	Founder 1 $\times$ Founder $\mathrm {n_f}$	1	2	0	2	1	0	.	0
	Founder 1 $\times$ Founder $\mathrm {n_f}$	2	2	0	0	0	0	.	1
	$\vdots$	$\vdots$	$\vdots$	$\vdots$	$\vdots$	$\vdots$	$\vdots$	$\ddots$	$\vdots$
	Founder 1 $\times$ Founder $\mathrm {n_f}$	$\mathrm {n_1}$	2	0	1	0.5	0	.	0.5
B	Founder 2 $\times$ Founder $\mathrm {n_f}$	1	0	0	0	0	0.5	.	0.5
	Founder 2 $\times$ Founder $\mathrm {n_f}$	2	0	0	0	0	0.5	.	0.5
	$\vdots$	$\vdots$	$\vdots$	$\vdots$	$\vdots$	$\vdots$	$\vdots$	$\ddots$	$\vdots$
	Founder 2 $\times$ Founder $\mathrm {n_f}$	$\mathrm {n_2}$	0	0	0	0	0.5	.	0.5

For a particular cross, marker genotypes of every line are compared with those of the founders to determine the founder probabilities. Example A illustrates a case where founders differ in their genotype, hence lines segregate for that marker and probabilities for each line can be inferred. On the contrary, example B shows a case where the marker is not segregating and lines have equal chance to inherit an allele from either of the parents and zero probability for the remaining founders

### Phenotyping

Two field experiments were conducted at the Queensland Government Department of Agriculture and Fisheries, Hermitage Research Facility, Warwick, Australia (WAR; $28.21^\circ$ S $152.10^\circ$ E, 480 m above sea level). The experiments correspond to the two winter seasons of 2015 and 2016 and were named WAR15 and WAR16. MR-NAM lines were grown following partially replicated designs (Cullis et al. 2006) with an average replication level of 1.40 and 1.46 in WAR15 and WAR16, respectively.

The trial layout was composed of plots arranged in 38 rows by 36 ranges in WAR15 and 63 rows by 20 ranges in WAR16. In both experiments, plot size was 2 m $\times$ 4 m with row spacing of 25 cm and a target population density of 100 plants/m$^2$. Further attributes of the two experimental environments were provided in Christopher et al. (2021), Table 1. QTL analysis for other traits has been reported elsewhere (Christopher et al. 2021) and here the focus is on plant height after flowering, measured to the top glume (cm).

Genotypes tested in WAR15 and WAR16 included the MR-NAM lines at F4:5 and F4:6, respectively, donor and reference parents, and other experimental and standard wheat lines. MR-NAM lines tested in WAR16 were a subset of those tested in WAR15 (Table 2). In WAR16, family Mace/ZWW10.50 and all families with Scout as the reference parent were poorly represented hence they were excluded from the association study performed for that year.

**Table 2:** Incomplete crossing scheme for the wheat MR-NAM population considered at each field experiment (WAR15 and WAR16)

Donors	WAR15 reference parent			WAR15 total	WAR16 reference parent		WAR16 total
	Mace$^{*}$	Scout$\dag$	Suntop$\dag$		Mace$^{*}$	Suntop$\dag$	
Dharwah dry$\dag$	43	50	42	135	37	32	69
Drysdale$^{*}$	45	42	50	137	45	46	91
EGA Gregory$\dag$			37	37		37	37
EGA Wylie$\dag$			37	37		30	30
FAC10.16$\dag$			39	39		39	39
SB062$\dag$	44	40	51	135	40	49	89
SeriM82$\dag$	42	50	46	138	38	44	82
UQ114$\dag$		42	40	82		39	39
Westonia$^{*}$	33			33	31		31
ZWB10.37$\dag$			34	34		32	32
ZWW10.50$\dag$	38			38			
Total	245	224	376	845	191	348	539

The number of recombinant inbred lines (RIL) considered per family and experiment is given. For the founders, the status of the dwarfing genes is provided as follows: $\dag$ indicates presence of the Rht-B1b dwarfing allele and $^{*}$ indicates presence of Rht-D1b dwarfing allele

### NAM simulated data

To create a NAM population with realistic marker profiles while the computational time for each simulation remains low, a subset of the DArT wheat consensus map (DArT 2018) was considered, as well as, a subset of founders to generate the crosses. Specifically, Suntop was selected as the reference parent to be crossed with 5 diverse donors (SeriM82, ZWB10.37, Drysdale, Westonia, and Dharwah dry). The linkage map for the simulation was a subset of the DArT consensus map (“Markers and map” section), consisting of seven LGs (1B to 7B) and the unique positions corresponding to those markers where segregation was expected in the NAM population, i.e., at least one donor was different to the reference parent at that position. This left seven LGs with between 77 and 223 unique positions and a total of 895 markers (Supplementary Fig. S1). The simulated NAM population consisted of five families of 100 RILs, corresponding to each of the biparental crosses between Suntop and the five donors hence a total of 500 lines.

The marker data for the NAM lines was simulated using R-qtl package (Broman et al. 2003). First, the RILs were generated at each biparental cross of donor and reference parent. Then, based on the founder marker data available, the data was modified to maintain segregation only where expected for each biparental cross.

The phenotypic data was simulated under two different scenarios to assess the Type I error rate and the power of the proposed methodology. The first scenario aimed to estimate the Type I error rate, i.e., the probability to detect at least one QTL when none existed. Phenotypic data with no QTL was generated assuming an experiment with two replicates following a simple model of the form:

1 $$\begin{aligned} y_{ir} = \mu + u_{\mathrm{g}_i} + e_{ir} \end{aligned}$$

where $y_{ir}$ represents the phenotypic value for line *i* ($i = 1, 2, ..., 500$) and replicate *r* ($r = 1, 2$), $\mu$ represents the overall mean, $u_{\mathrm{g}_i}$ represents the polygenic random effect for line *i*, and $e_{ir}$ represents the residuals. For the simulation, it was assumed that $\mu = 7,\; u_{\mathrm{g}_i} \sim N(0, 0.5)$, and $e_{ir} \sim N(0, 1)$. A total of 500 simulations were generated for the NAM population where no QTL effects were present.

The second scenario for simulation of phenotypic data considered the presence of eight QTL, their position and size are provided in Table 3. Phenotypic data with the eight QTL effects was generated assuming an experiment with two replicates following an extension of the previous simulation model to incorporate the QTL effect as follows:

2 $$\begin{aligned} y_{ir} = \mu + \sum _{j=1}^{{8}}\sum _{l=1}^{{6}}{{p}_{{i}_{jl}}{a}_{jl}} + u_{\mathrm{g}_i} + e_{ir} \end{aligned}$$

where $${a}_{jl}$$ are the effects for QTL *j* ($$j = 1, 2, ...8)$$ and founder *l* ($$l = 1, 2, ..., 6)$$ given in Table 3 and $${p}_{{i}_{jl}}$$ is the probability of line *i* receiving alleles from founder *l* at QTL *j*. Note that $$\sum _{l=1}^{{6}}{{p}_{{i}_{jl}}=1}$$. Again, a total of 500 simulations were generated for the NAM population.

**Table 3:** QTL parameters for the simulations assessing power

QTL	Distance (cM)	LG	Marker	Founder					
				Suntop	Seri.M82	ZWB10.37	Drysdale	Westonia	Dharwah dry
QTL1	94.39	1B	LG.1B.53	0.4	− 0.2	− 0.2	0	0	0
QTL2	17.84	2B	LG.2B.8	0.5	− 0.1	− 0.1	− 0.1	− 0.1	− 0.1
QTL3	19.00	3B	LG.3B.20	0.4	0	0	− 0.4	0	0
QTL4	62.27	4B	LG.4B.60	0	− 0.6	0	0	0	0.6
QTL5	60.11	5B	LG.5B.148	0.2	0	0	− 0.1	0	− 0.1
QTL6	57.39	6B	LG.6B.63	0.2	− 0.04	− 0.04	− 0.04	− 0.04	− 0.04
QTL7	101.51	7B	LG.7B.102	0.2	0	0	0	0	− 0.2
QTL8	161.57	1B	LG.1B.135	0	− 0.3	0	0	0.3	0

Eight QTL were positioned across 7 linkage groups (LGs) and the effects for each QTL and founder alleles were specified

## Methods

The methodology presented here to perform a whole-genome analysis for NAM populations, namely WGNAM, is an extension of the linear mixed model that forms the basis of the whole-genome analysis for MAGIC populations introduced by Verbyla et al. (2014b), MPWGAIM. The following sections describe how the MPWGAIM model was modified to incorporate the NAM population structure, revisit the putative QTL selection process, and review the final model assessments. Finally, the performance of WGNAM was assessed with the simulated data to estimate the false positive rate and the power to detect QTL and to compare the results with other methods of reference.

### The linear mixed model

Genetic studies involve the collection of phenotypic data based on experimental designs. An efficient analysis of that data is often performed in a linear mixed model framework since it allows the inclusion of fixed or random effects that are present in the trial. The model fitted to the data is of the form:

3 $$\begin{aligned} \mathbf {y} = \mathbf {X}\varvec{\tau } + \mathbf {Z}_{\mathrm {0}}\mathbf {u}_{\mathrm {0}} + \mathbf {Z}_{\mathrm{g}}\mathbf {u}_{\mathrm{g}}+\varvec{\epsilon } \end{aligned}$$

where $\mathbf {y}$ is the vector $N \times 1$ of all trait data points (*N*) and $\mathbf {X}$ and $\mathbf {Z}_{{0}}$ are the incidence matrices of dimensions $N \times n_\tau$ and $N \times n_{u_0}$ corresponding to the fixed effects $\varvec{\tau }$ and the non-genetic random effects $\mathbf {u}_{{0}}$, respectively. These two effects reflect the experimental design of the trial and any other variation that requires modeling (Smith et al. 2005, 2006). The random effects $\mathbf {u}_{{0}}$ and the residual vector denoted by $\varvec{\epsilon }$ ($N \times 1)$ are assumed to be independent, normally distributed with mean zero and variance-covariance matrices $\mathbf {G}_{\mathrm {0}}$ and $\mathbf {R}$, respectively. Properly modeling non-genetic effects will help to correctly determine the genetic effects $\mathbf {u}_{\mathrm {g}}$, which are the main focus in this study (Verbyla et al. 2014b).

Suppose there are ${n_g}$ lines of interest, the incidence matrix $\mathbf {Z}_{\mathrm{g}}$ of dimension $N \times {n_g}$ assigns to each observation the appropriate random genetic effect from $\mathbf {u}_{\mathrm{g}}=[ u_{\mathrm{g}_i}]$ of dimension ${ {n_g} \times 1}$. The simplest model that could be considered for the genetic effects for a line $i\;(i = 1, 2,\dots , {n_g})$ is the so-called infinitesimal or polygenic model for which:

4 $$\begin{aligned} u_{\mathrm{g}_i} = u_{\mathrm{p}_i} \end{aligned}$$

The matrix form of model (4) is $\mathbf {u}_{\mathrm{g}} = \mathbf {u}_{\mathrm{p}}$ where $\mathbf {u}_{\mathrm p} \sim N(\mathbf {0},\sigma ^2_{ p}\mathbf {I}_{ {n_g}})$, i.e., polygenic effects are assumed to be zero centered and independent although pedigree information could also be included (Oakey et al. 2007).

Association studies rely on the integration of the population structure and molecular marker data into the analysis of the trait of interest. Thus, an appropriate specification of the genetic effects should be accomplished through models more complex than (4).

### The basis of the WGNAM model

Suppose there are ${n_m}$ molecular markers in total across all the linkage groups or chromosomes in the genome. A possible (but unrealistic) integration of model (4) with these markers is one that allows for a QTL at the position of each marker on the genetic map:

5 $$\begin{aligned} {u}_{\mathrm{g}_i} = \sum _{j=1}^{{n_m}}{z}_{\mathrm{m}_{ij}}{m}_j + {u}_{\mathrm{p}_i} \end{aligned}$$

where ${z}_{\mathrm{m}_{ij}}$ is the known marker genotype that corresponds to line *i* and marker at position *j* on the genetic map, coded as the number of copies of a given allele (0, 1 or 2), ${m}_j$ is the random effect of the marker at position $j\,(j = 1, 2,\dots , {n_m})$ where ${m}_j\sim N(0,\sigma ^2_{ m})$, and the last component (${u}_{\mathrm{p}_i}$) is the polygenic effect that allows for possibly a large number of small QTL that cannot be detected individually (Verbyla et al. 2012).

Consider now the population structure, and assume there are ${n_f}$ founders. The expression of putative QTL could depend on the founders and this can be modeled as follows:

6 $$\begin{aligned} {u}_{\mathrm{g}_i} = \sum _{j=1}^{{n_m}}\mathbf {q}^T_{{i}_{j}}\mathbf {m}_j + {u}_{\mathrm{p}_i} \end{aligned}$$

where $\mathbf {m}_j$ is the ${n_f} \times 1$ vector of ${n_f}$ founder allele effects at position *j* and $\mathbf {q}_{{i}_{j}}$ is the ${n_f}\times 1$ vector of founder alleles for line *i* and marker at position *j*. For a given line in the NAM population, the elements of $\mathbf {q}_{{i}_{j}}$ indicate the number of alleles coming from each founder thus, most of the elements are zero except for up to two elements corresponding to the two parents of that line and $\sum _{l=1}^{ {n_f}}{q}_{ijl} = 2$.

The origin of the alleles at the putative QTL is not always certain then, neither are the elements in $\mathbf {q}_{{i}_{j}}$ in model (6). Following Verbyla et al. (2014b), the way to account for this uncertainty is to use the probabilities of inheriting founder alleles, such as the ones described in “MR-NAM founder probabilities calculation” section. This is the major contribution of the MAGIC method for structured populations, and therefore the major component that needs to be modified to take into account the population structure of the NAM populations.

Let $\mathbf {p}_{{i}_{j}}$ be the ${n_f} \times 1$ vector of founder probabilities for line *i* and marker at position *j*. Note that $\sum _{l=1}^{{n_f}}{p}_{ijl} = 1$ since they are probabilities now instead of the number of alleles.

The WGNAM model considering probabilities of inheriting founder alleles is the basis of this association study and has the form:

7 $$\begin{aligned} {u}_{\mathrm{g}_i} = \sum _{j=1}^{{n_m}}\mathbf {p}^T_{{i}_{j}}\mathbf {a}_j + {u}_{\mathrm{p}_i} \end{aligned}$$

where $\mathbf {a}_j$ is the ${n_f} \times 1$ vector of founder specific effects at position *j* which are assumed to be random, independent and identically distributed $\mathbf {a}_j{\sim } N(\mathbf {0},\sigma ^2_{ a}\mathbf {I}_{ {n_f}})$.

The determination of the probabilities $\mathbf {p}_{{i}_{j}}$ was discussed in detail for MAGIC population by Verbyla et al. (2014b). In “MR-NAM founder probabilities calculation” section, one approach is presented to determine the probabilities of inheriting founder alleles in a NAM population.

### Putative QTL selection for WGNAM

The WGNAM analysis builds the final model by selecting a set of the potential QTL from the total number of markers. The selection process implemented here corresponds to that proposed by Verbyla et al. (2014b). First, a test is performed to check if there is evidence of at least one putative QTL. The questions that follow are how many putative QTL are present and where they are positioned in the genetic map. These questions are addressed following a forward variable selection approach. Intrinsically, it is an iterative process that first performs the test for a putative QTL described below, then identifies the most important marker position, and repeats this process until there is no longer sufficient evidence of additional QTL.

#### Test for a putative QTL

This first step consists of contrasting two models that result from fitting the linear mixed model (3) where the genetic effects are modeled *i*) only by polygenic effects as in (4) and *ii*) by molecular markers and polygenic effects as in (7). Both models are then compared to test the hypothesis $H_{{0}}: \sigma ^2_{ a}=0$, i.e., if there is sufficient variance of marker effects to warrant the selection of a putative QTL. The conducted test is a likelihood ratio test where the null distribution is a mixture of a chi-squared distributions, namely $0.5\chi ^2_{{0}} + 0.5\chi ^2_1$ (Stram and Lee 1994).

#### Selection of the first putative QTL

The selection of the first putative QTL (iteration 1), is based on the outlier statistic for every marker position using model (7). The foundation of the outlier statistic for a position $j'$ is the alternative outlier model below:

8 $$\begin{aligned} \begin{aligned} {u}_{\mathrm{g}_i}&= \sum _{j=1}^{{n_m}}\mathbf {p}^T_{{i}_{j}}\mathbf {a}_j + \mathbf {p}^T_{{i}_{j'}}\varvec{\delta }_{j'} + {u}_{\mathrm{p}_i} \\&= \sum _{\underset{j \ne j'}{j=1}}^{ {n_m}}\mathbf {p}^T_{{i}_{j}}\mathbf {a}_j + \mathbf {p}^T_{{i}_{j'}}\Big (\mathbf {a}_{j'} + \varvec{\delta }_{j'}\Big ) + {u}_{\mathrm{p}_i} \end{aligned} \end{aligned}$$

where $\varvec{\delta }_{j'}$ is a ${n_f} \times 1$ vector corresponding to position $j'$ that is assumed to be $\varvec{\delta }_{j'}\sim N(\mathbf {0},\sigma ^2_{{a}_{j'}}\mathbf {I}_{ {n_f}})$. The alternative outlier model inflates the QTL effects at position $j'$ by $\varvec{\delta }_{j'}$ while the outlier model under the null hypothesis, that is with $\sigma ^2_{{a}_{j'}}=0$, is equivalent to (7). Calculating the outlier statistic for every position seems to imply that many models need to be fitted (one per marker). However, a procedure based on a score statistic for testing $H_{{0}}: \sigma ^2_{{a}_{j'}}=0$ only relies on the null model, hence, only model (7) would need to be fitted (Verbyla et al. 2012).

If $\tilde{{a}}_{jl}\,(l=1,2,\dots ,{n_f})$ represents the best linear unbiased predictor (BLUP) of ${a}_{jl}$, i.e., the size of the potential QTL effect for founder *l* at position *j*, the outlier statistic is given by:

9 $$\begin{aligned} {t}^2_{j} = \frac{\sum _{l=1}^{{n_f}}\tilde{{a}}^2_{jl}}{\sum _{l=1}^{{n_f}}\mathrm{var}\left( \tilde{{a}}_{jl}\right) } \end{aligned}$$

That is, the outlier statistic sums over the squared effects of all the founders at a specific position *j* in the genome. Positions with important effects will result in large values of the outlier statistic. Hence, the largest value of the outlier statistic among all the marker positions in the genome is selected as the first putative QTL.

#### Selection of additional QTL

To test the presence of a second QTL, the process is equivalent to the one described above but this time, the models to be compared by the likelihood ratio test include the first putative QTL already detected.

Assume that ${n_s}$ is the number of putative QTL found and *S* is the subset of all their positions, the general form of the two models involved in that test are:

10 $$\begin{aligned} {u}_{\mathrm{g}_i} = \sum _{j \in S}^{}\mathbf {p}^T_{{i}_{j}}\mathbf {a}_{j} + {u}_{\mathrm{p}_i} \end{aligned}$$

and,

11 $$\begin{aligned} {u}_{\mathrm{g}_i} = \sum _{j \in S}^{}\mathbf {p}^T_{{i}_{j}}\mathbf {a}_{j} + \sum _{j \not \in S}^{}\mathbf {p}^T_{{i}_{j}}\mathbf {a}_j + {u}_{\mathrm{p}_i} \end{aligned}$$

where $\mathbf {a}_{j} \sim N(\mathbf {0},\sigma ^2_{{a}_j}\mathbf {I}_{ {n_f}})$ if $j \in S$ and $\mathbf {a}_{j} \sim N(\mathbf {0},\sigma ^2_{ a}\mathbf {I}_{{n_f}})$ if $j \not \in S$. Note that there is one variance component for each selected putative QTL ($\sigma ^2_{{a}_j}$) and a common variance ($\sigma ^2_{ a}$) for the effects of all remaining positions where no putative QTL were found.

### Final WGNAM model

Once the selection process terminates, the final WGNAM model is the linear mixed model (3) with (11), which considers all the putative QTL found as random effects. Additional assessments of the final model are considered to better understand the QTL size effects (Verbyla et al. 2012, 2014b).

Essentially, QTL effects are estimated by the BLUP of $\mathbf {a}_{j}\;j\in S$, i.e., $\tilde{\mathbf {a}}_{j}\;j\in S$, which will inform about the size of the effects of the putative QTL for each individual founder. The approach to measure the strength of putative QTL effect $\mathbf {a}_{j}$ depends on the normality assumptions of the linear mixed model which leads to the conditional distribution:

12 $$\begin{aligned} \mathbf {a}_{j}|\mathbf {y}_2\sim N(\tilde{\mathbf {a}}_{j}, \mathbf {\Sigma }_{\mathrm{PEV},j}) \end{aligned}$$

where $\mathbf {y}_2$ is the component of data free of fixed effects (Verbyla 1990), the mean and the variance are, respectively, the BLUP of $\mathbf {a}_{j}$ and the prediction error variance (PEV) $\mathbf {\Sigma }_{\mathrm{PEV},j}$. Defining $\mathbf {\Sigma }^{-}$ a generalized inverse of $\mathbf {\Sigma }$, a measure of the strength of a putative QTL is given by the following overall probability (Verbyla et al. 2014b):

13 $$\begin{aligned} {p}_j = P\left( \Big (\mathbf {a}_j -\tilde{\mathbf {a}}_j\Big )^{T} \mathbf {\Sigma }^{-}_{{PEV},j} \Big (\mathbf {a}_j -\tilde{\mathbf {a}}_j\Big ) > \tilde{\mathbf {a}}_j^{T} \mathbf {\Sigma }^{-}_{{PEV},j} \tilde{\mathbf {a}}_j \right) \end{aligned}$$

or equivalently, the LOGP score: LOGP$_j = -\hbox {log}_{10}({p}_j)$.

### WGNAM performance evaluation

The simulated NAM data described in “NAM simulated data” section which covered two scenarios (absence/presence of QTL) served to study the Type I error rate and power of WGNAM. Furthermore, results were compared with two multi-QTL approaches: a GWAS approach and a NAM specific approach. The nominal Type I error rate was set at $\alpha = 0.05$ for WGNAM but a Bonferroni correction for multiple testing was implemented in the alternative methods ($\alpha _\mathrm{bonf} = 0.05/896$), otherwise all the simulations had at least one false positive (results not shown).

The GWAS method implemented in this study was MLMM (Segura et al. 2012). The MLMM approach is a two-stage GWAS method so, phenotypic data was averaged across replicates for each simulation. The kinship matrix was utilized to correct for the population structure. To obtain a multi-QTL model in each simulation, a forward selection process was followed with a threshold set at $\alpha _\mathrm{bonf}$ and the best model was selected using the extended Bayesian Information Criteria (eBIC)  as proposed by the MLMM authors.

The NAM specific approach implemented here was the multi-QTL model (MQE) from the MPP methodology (Garin et al. 2017). Likewise MLMM, MPP is a two-stage method so, phenotypic data was averaged across replicates for each simulation. The MPP method estimates parental effects by calculating IBD probabilities utilizing the R-qtl package (Broman et al. 2003). With a threshold equivalent to $\alpha _\mathrm{bonf}$ a subset of detected QTL was considered first and then a forward selection process was followed to build the multi-QTL model (setting a minimum distance between two QTL of 20 cM).

The simulation study using WGNAM was carried out with the same IBD probabilities utilized in the MPP approach, given that the WGNAM method is flexible about the IBD calculation method and this will facilitate the comparison. For the power study, a QTL was considered to be detected if it was 10 cM either side of the true QTL. This was in agreement with the minimum distance between QTL define for the MPP method.

### Computation

All the analyses were performed in R (R Core Team 2019). The WGNAM approach has been implemented using the package wgnam available at https://github.com/ValeriaPaccapelo/wgnam. The main dependencies on R packages are mpwgaim (Verbyla et al. 2014b) and asreml (Butler 2009). In particular, it is the power of asreml that allows the complex models to be fitted. The MLMM approach was implemented via the package mlmm.gwas (Bonnafous et al. 2019), whereas the MPP approach was implemented using the mppR package (Garin et al. 2018).

## Results

### WGNAM performance with NAM simulated data

In the first scenario, the NAM simulated data in the absence of QTL, the Type I error rate was estimated from 500 simulations implementing WGNAM with a nominal Type I error rate of $\alpha = 0.05$, whereas for the methods MLMM and MPP the nominal threshold was based on the Bonferroni correction. The probabilities of finding at least one false positive QTL were 0.044, 0.092, and 0.01 for the WGNAM, MLMM, and MPP, respectively. These figures indicate that WGNAM does control the false positive rate without implementing any multiple testing correction under this scenario of no QTL. The Bonferroni correction was not enough for the MLMM approach given the realized Type I error rate almost doubled the expected value of 0.05 while was too conservative for the MPP method. The mean number of detected QTL were 0.052, 0.10, and 0.01 for the methods WGNAM, MLMM, and MPP, respectively. These results showed that there were some simulations where more than one false QTL was detected for the methods WGNAM and MLMM but not for MPP. The latter prevents the search of an additional QTL given a certain window (20 cM in this study, i.e., 10 cM each side of the detected QTL).

The second scenario considered the presence of QTL effects and applied the same nominal thresholds described above. The estimation of the power of the three methods to detect each of the eight QTL across the 500 simulations is presented in Table 4. All the methods were consistent for QTL1, QTL2, and QTL3, showing a rate above 0.80. Conversely, the detection rate was low for QTL8, failing to detect it. For the remaining QTL, the WGNAM method presented the highest rate of detection (varying from 0.378 to 0.646) and the MPP method was only superior to the MLMM approach for QTL4. The resolution was also evaluated and for those combinations of method-QTL with a detection rate above 50%, the distance between the true QTL and the detected one was less than 1 cM for all the methods.

Furthermore, within the scenario with QTL effects, the Type I error rate was estimated for the three methods, across all the LGs (Table 4). The WGNAM method presented a high rate of false positives with at least one false positives 60% of the times and on average, 1.050 false positives per simulation. The MLMM approach presented false positive rate of 0.232 and the MPP was even better with a rate of 0.092.

**Fig. 1:**
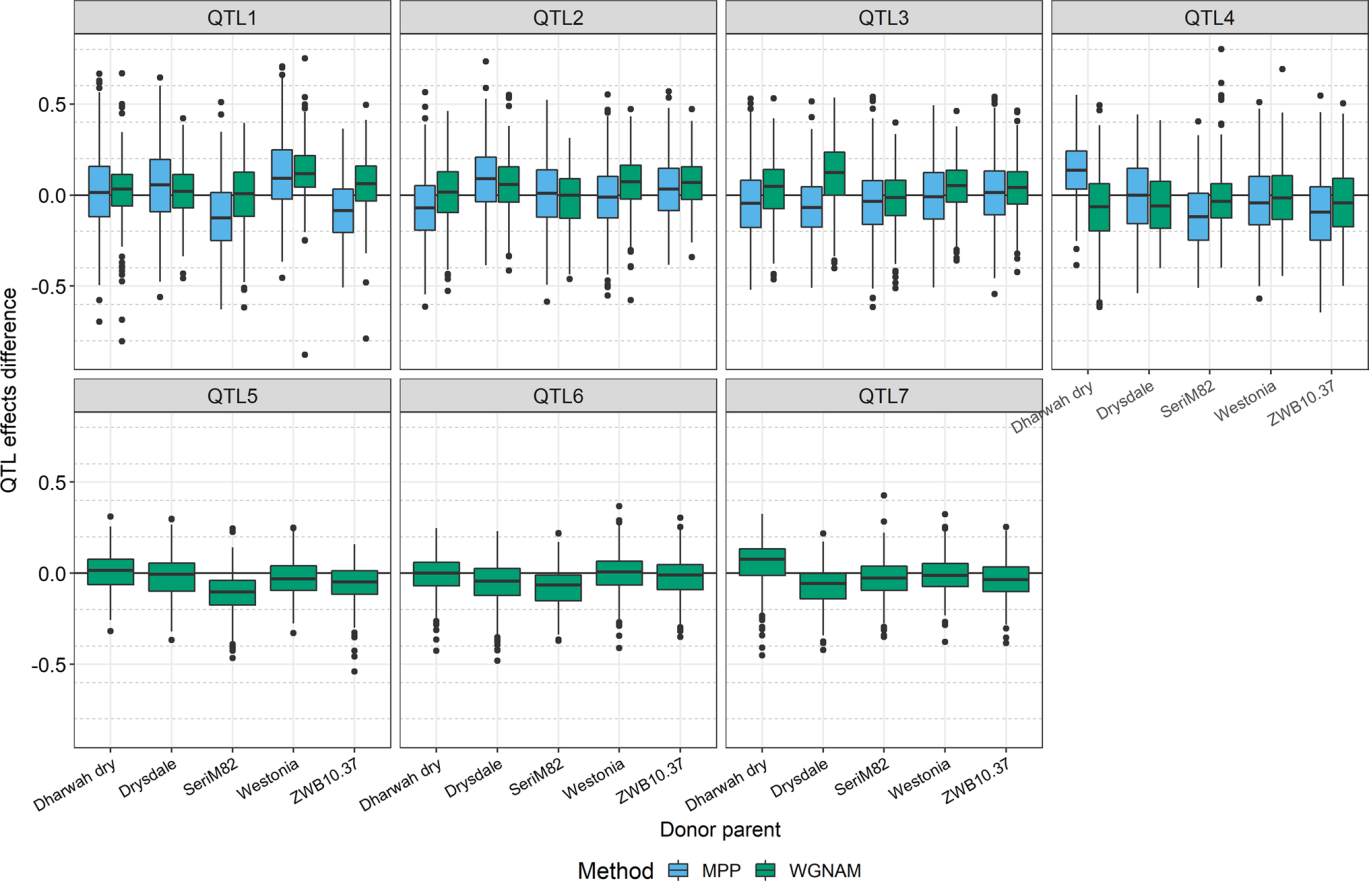
Distribution of the difference between the QTL effect estimations and the true values in the simulation study for a NAM population applying WGNAM and MPP methods. Distributions are shown for each founder and grouped by QTL. Results are not shown for the MPP approach when presented a detection power below 15%

The QTL effects were estimated for each simulation when detected under the second scenario. The WGNAM method produces zero centered predictions and the prediction represents the effect of having the same genotype that a given founder at that position (probability of 1, see Eq. 7), in agreement with Table 3. The MPP method estimates allele effects relative to the reference parent (with allele effect set to zero); furthermore, the model considers allele number rather than probabilities, so the effects need to be multiplied by two if the line is homozygote. To compare methods, MPP estimations were multiplied by two and centered for each simulation to obtain estimations for all the founders that are centered around zero. Figure 1 shows the difference between the estimated QTL effects and the true values for QTL1 to QTL4 for WGNAM and MPP methods given that both methods showed reasonable power values (Table 4), but for that reason, QTL5 to QTL7 show results for WGNAM only. For QTL1 to QTL4, the WGNAM effects were, in general, less variable than MPP effects, whereas the difference from zero sometimes depended on the QTL and founder. For QTL1 and QTL4, where the power of the MPP is lower than WGNAM, some of the founder effects are more distant from zero for the MPP method. For QTL5 to QTL7 where QTL effects had smaller size compared with QTL1 to QTL4, the WGNAM method shows some bias for some of the founders but still was able to detect these QTL with a better rate than the MPP method (Table 4).

The MLMM estimations of QTL effects could only be compared to the true values when all the effects for the donors were the same (QTL2 and QTL4). Having a very strong power to detect the QTL2, the MLMM mean estimation was 0.57 for a genotype equivalent to Suntop (with a true value of 0.50) and 0.056 for any homozygote genotype that was not Suntop, i.e., equivalent to any donor parent (with true value of 0.10). For QTL4 with smaller effects, the MLMM method presented a lower detection rate (0.44) and greater bias (for Suntop equivalent genotype the estimation was 0.36 vs the true value of 0.20 and for the alternative homozygote genotype the estimation was 0 vs 0.04).

**Table 4:** Proportion of QTL detection rates across 500 simulations applying three different approaches: whole-genome nested association mapping (WGNAM), multi-locus mixed model (MLMM), and multi-parent population (MPP)

QTL	Method		
	WGNAM	MLMM	MPP
QTL1	0.962	0.968	0.798
QTL2	1.000	1.000	0.984
QTL3	0.986	0.808	0.922
QTL4	0.646	0.004	0.532
QTL5	0.378	0.106	0.072
QTL6	0.620	0.440	0.104
QTL7	0.556	0.240	0.074
QTL8	0.058	0.040	0.014
False QTL (Type I rate)	0.600	0.232	0.092
False QTL (Mean number)	1.050	0.714	0.098

For the eight QTL, a QTL was considered “detected” if the position of the detected marker was within 10 cM from the true QTL position otherwise it was considered a false QTL and the false positive rate (Type I error) and the mean number of detections were provided

### Phenotypic assessment in an MR-NAM population

**Fig. 2:**
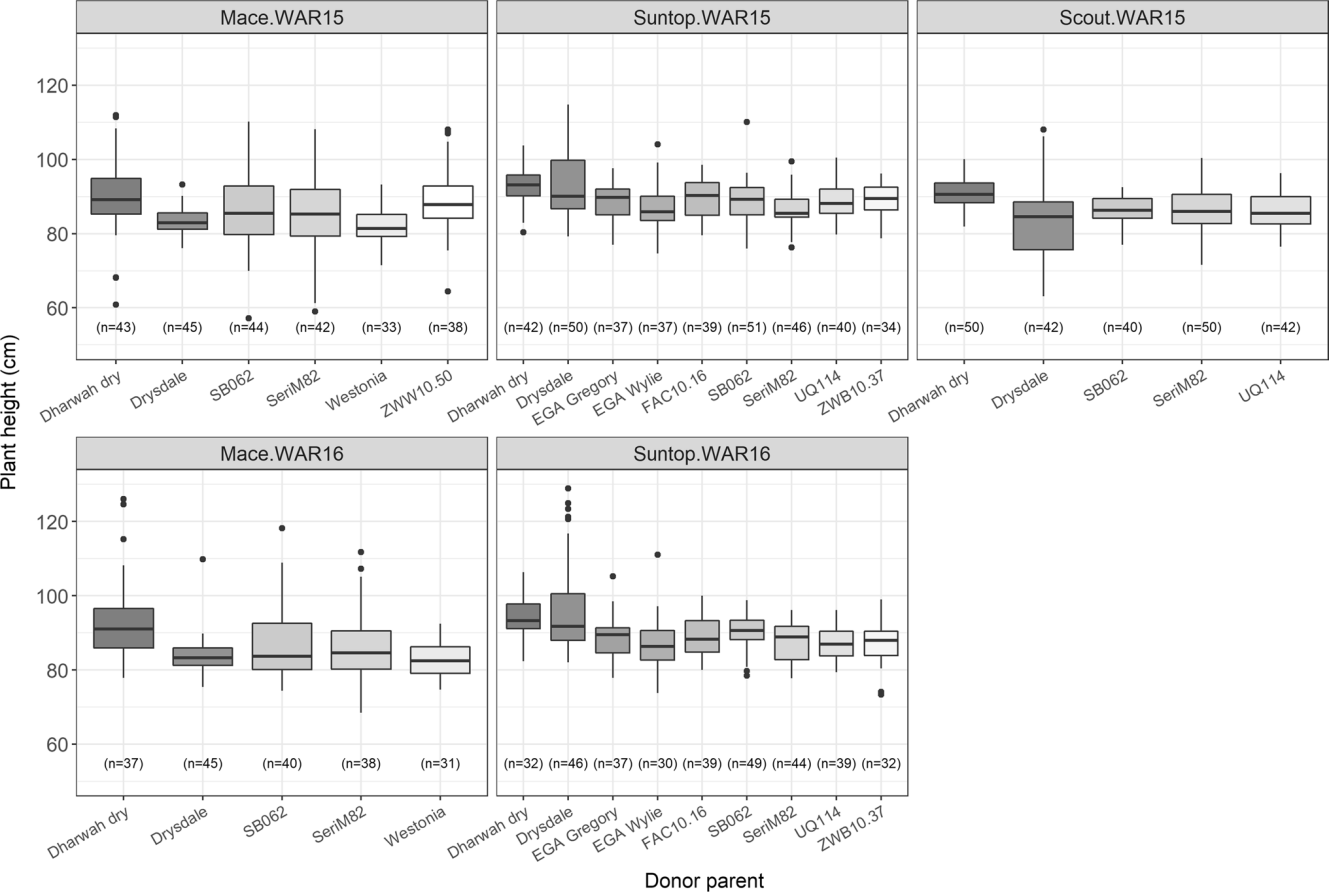
Distribution of the plant height best linear unbiased predictions (BLUPs) for each family in the multi-reference nested association mapping (MR-NAM) population at each experiment. Family distributions are shown for each donor and grouped by reference parent (Mace, Suntop and Scout) and experiment (WAR15 and WAR16). BLUPs are the result of the phenotypic analysis using the base linear mixed model. Number of lines per family *n* tested in each trial are provided in brackets. Data for Scout families was not included in the analysis for WAR16

The analysis of plant height data at each field trial started with a phenotypic analysis using the base linear mixed model (model 3). This accounted for experimental design factors (replicate blocks), fixed effects for lines that were not part of the MR-NAM population (including standard wheat lines, founders, etc.) and random genotype effects for MR-NAM lines with molecular data. The residuals were considered to be correlated and followed a multiplicative autoregressive model of first order in the row and column directions in WAR15 but only in the row direction in WAR16. For illustrative purposes, BLUPs were obtained from the base linear mixed model for all MR-NAM lines in each experiment and are summarized in Fig. 2. Despite the moderate selection for a semi-dwarfing phenotype, considerable variation for plant height remained within the population. Overall, similar plant height was observed between years for those families tested at both environments. In the case of WAR15, all families within each of the three NAM populations showed variability for plant height. Moreover, donors behaved similarly within each background. For instance, families with Dharwah dry parentage had generally taller lines compared to Drysdale. Overall, there were no clear differences among the reference backgrounds. However, when comparing families derived from particular donors some differences became more evident. For example, Drysdale tended to have taller plants when crossed to Suntop compared to Scout and Mace. In WAR16, only Mace and Suntop families were grown. Similarly to WAR15, differences due to donors were observed as well as different performance for the same donor in different reference backgrounds (Fig. 2).

### The putative QTL search

**Fig. 3:**
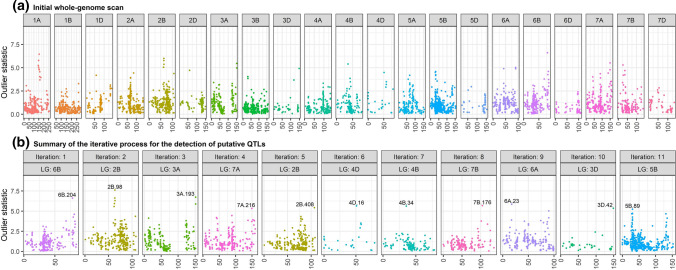
Summary of the putative QTL search performed for plant height in WAR16. Values of the outlier statistic are plotted against the distance (cM) within linkage group (LG) according to the consensus map (DArT 2018). (a) Whole-genome values of the outlier statistic for the first iteration. (b) Whole-genome values of the outlier statistic for the remaining iterations, only representing the values for the LG where the putative QTL was identified. The outlier statistic value for the putative QTL is tagged with the identified position (LG and marker order within LG)

To illustrate the putative QTL search performed by WGNAM, Table 5 presents a summary of the iterative process carried out for plant height in WAR16. It summarizes the iterative process to test the presence of at least one (or more) QTL, and identifies the position of the putative QTL via the outlier statistic. The outlier statistic for the first iteration indicated the presence of at least one QTL for plant height (*p*-value = 5.55E − 17). In order to identify the position, the outlier statistic was calculated for every possible position across the genome (top panel of Fig. 3). The maximum value of the outlier statistic in the first iteration was identified on LG 6B, position 204 (LG.6B.204) and corresponded to the marker at a distance of 78.29 cM on LG 6B of the consensus map (DArT 2018). This putative QTL was incorporated into the linear mixed model, and then iteration 2 indicated presence of an additional QTL. The values of the outlier statistic in the second iteration were calculated across the whole-genome to search for the maximum; however, for simplicity Fig. 3 shows the values corresponding to the LG 2B where the second putative QTL was identified. The process continued until there was no significant evidence of additional QTL (*p*-value $> 0.05$). Hence, 11 putative QTL were identified for plant height in WAR16.

Similarly, a WGNAM iteration process was performed for plant height in WAR15. Since it comprised 27 iterations (26 putative QTL), the details of this whole process are not shown.

**Table 5:** Summar﻿y of the iterative process behind the association study performed for plant height in WAR16

Iteration	Model log-likelihood		Likelihood ratio	*p*-value	Outlier statistic	Identified position	Linkage group	Distance (cM)
	Reduced	Complete						
1	$-$ 1988.64	$-$ 1953.61	70.06	5.55E $-$ 17	5.43	LG.6B.204	6B	78.29
2	$-$ 1966.13	$-$ 1948.96	34.35	2.31E $-$ 09	7.61	LG.2B.98	2B	63.52
3	$-$ 1946.44	$-$ 1942.66	7.57	0.0030	6.73	LG.3A.193	3A	151.45
4	$-$ 1936.88	$-$ 1935.46	2.83	0.0462	5.35	LG.7A.216	7A	149.56
5	$-$ 1932.19	$-$ 1930.62	3.13	0.0384	5.62	LG.2B.408	2B	107.01
6	$-$ 1928.26	$-$ 1925.83	4.87	0.0136	5.64	LG.4D.16	4D	50.96
7	$-$ 1923.32	$-$ 1921.25	4.14	0.0209	5.23	LG.4B.34	4B	35.55
8	$-$ 1918.95	$-$ 1917.05	3.81	0.0255	5.85	LG.7B.176	7B	104.50
9	$-$ 1916.12	$-$ 1914.58	3.08	0.0396	6.61	LG.6A.23	6A	17.57
10	$-$ 1912.65	$-$ 1911.11	3.09	0.0395	5.33	LG.3D.42	3D	149.01
11	$-$ 1910.43	$-$ 1909.06	2.75	0.0488	5.68	LG.5B.89	5B	30.46
12	$-$ 1907.53	$-$ 1906.55	1.96	0.0808	–	–	–	–

For each iteration, the log-likelihood of the fitted models, likelihood ratio test statistic value, associated probability, outlier statistic value, identified position, and distance are presented

### Assessment of the final model

After the search for putative QTL, further assessments of the final model can be performed to gain insight into the QTL effects of each founder for each putative QTL. For illustrative purposes, Table 6 presents the estimated founder specific sizes for plant height in WAR16 for the first four putative QTL detected in the WGNAM iterative process (Table 5). In the final model, the first putative QTL detected on 6B at 78.29 cM showed a LOGP score of 2.74 accounting for a 17.61% of the plant height genetic variability of the MR-NAM population. Founder specific LOGP scores indicated that the sources of important QTL size corresponded to Suntop (Suntop genotype reduced plant height by 7.68 cm), and Drysdale and Mace (plant height increasing effects). Based on the marker genotype for the founders (Table 6) only populations with Drysdale (Drysdale marker genotype $= 0$) were expected to segregate. Also, Suntop and Mace both had the same genotype for this marker (marker genotype $= 2$) but opposite effects ($-7.68$ and 4.69, respectively), hence the importance of considering the allele origin in the model.

On LG 2B at 63.52 cM, the putative QTL had a LOGP score of 1.16, explaining 11.15% of the genetic variability overall. Suntop was the unique founder that increased plant height significantly (LOGP score = 2.28). The only populations expected to segregate were Drysdale and EGA Wylie both crossed to Suntop (Tables 2 and 6).

A putative QTL on 3A at 151.45 cM showed a LOGP score of 2.23, accounting for 3.07% of the genetic variance of the whole MR-NAM population (Table 6). Drysdale presented quite a strong size (LOGP score = 2.54) but as a donor parent gave origin to only 91 lines out of 539 in WAR16, with only a subset of these potentially carrying the Drysdale allele. Other donors that showed relatively strong effects were EGA Wylie and SB062. These were donor parents of 37 and 82 lines in the MR-NAM population (Table 2).

The remaining putative QTL in Table 6 was located on LG 7A. The putative QTL showed a LOGP score of 2.11, accounting for 9.52% of the genetic variability (Table 6). Regarding the founder specific effects, the founders that presented strong QTL size were Drysdale, Dharwah dry, SeriM82, and Mace. Note that, based on the marker genotype of the founders, only crosses with Mace are expected to segregate in the population and the donors just mentioned were crossed to Mace (SB062 and Westonia were also crossed to Mace but there was no evidence of strong effects of their genotypes) (Tables 2 and 6).

**Table 6:** Extract of the assessment of the putative QTL detected for plant height in WAR16. For the first four detected putative QTL, a summary of founder specific effects is provided (size, probability, and LOGP score) as well as overall effects (probability, percentage of genetic variance attributed to the putative QTL [%var] and LOGP score)

Identified position	Distance (cM)	Founder	Marker genotype	Founder specific			Overall		
				Size	Probability	Score	Probability	% var	Score
LG.6B.204	78.29	Dharwah dry	2	2.48	0.3131	0.50	0.0021	17.61	2.74
		Drysdale	0	4.42	0.0936	1.03			
		EGA gregory	2	$-$ 0.37	0.4722	0.33			
		EGA wylie	2	$-$ 0.51	0.4611	0.34			
		FAC10.16	2	0.17	0.4877	0.31			
		Mace	2	4.69	0.0956	1.02			
		SB062	2	0.57	0.4563	0.34			
		SeriM82	2	$-$ 1.62	0.3778	0.42			
		Suntop	2	$-$ 7.68	0.0161	1.81			
		UQ114	2	$-$ 1.02	0.4244	0.37			
		Westonia	2	$-$ 0.72	0.4455	0.35			
		ZWB10.37	2	$-$ 0.41	0.4693	0.33			
		ZWW10.50	2	0.00	0.5000	0.30			
LG.2B.98	63.52	Dharwah dry	0	1.52	0.3581	0.45	0.0693	11.15	1.16
		Drysdale	2	$-$ 1.57	0.2748	0.56			
		EGA gregory	0	$-$ 0.23	0.4794	0.32			
		EGA wylie	2	$-$ 1.94	0.2761	0.56			
		FAC10.16	0	0.10	0.4910	0.31			
		Mace	0	$-$ 2.72	0.1750	0.76			
		SB062	0	0.35	0.4671	0.33			
		SeriM82	0	$-$ 0.99	0.4073	0.39			
		Suntop	0	6.80	0.0057	2.28			
		UQ114	0	$-$ 0.62	0.4429	0.35			
		Westonia	0	$-$ 0.44	0.4597	0.34			
		ZWB10.37	0	$-$ 0.25	0.4775	0.32			
		ZWW10.50	0	0.00	0.5000	0.30			
LG.3A.193	151.45	Dharwah dry	2	0.85	0.3968	0.40	0.0067	3.07	2.23
		Drysdale	0	5.13	0.0037	2.54			
		EGA gregory	2	$-$ 0.13	0.4859	0.31			
		EGA wylie	0	$-$ 3.66	0.0692	1.16			
		FAC10.16	2	0.06	0.4935	0.31			
		Mace	2	$-$ 1.89	0.1348	0.87			
		SB062	0	2.74	0.0710	1.15			
		SeriM82	0	0.12	0.4749	0.32			
		Suntop	2	$-$ 1.03	0.2654	0.58			
		UQ114	2	$-$ 0.35	0.4587	0.34			
		Westonia	0	$-$ 1.34	0.2941	0.53			
		ZWB10.37	0	$-$ 0.51	0.4345	0.36			
		ZWW10.50	2	0.00	0.5000	0.30			
LG.7A.216	149.56	Dharwah dry	0	5.67	0.0309	1.53	0.0082	9.52	2.11
		Drysdale	0	$-$ 6.72	0.0097	2.05			
		EGA gregory	0	$-$ 0.29	0.4761	0.32			
		EGA wylie	0	$-$ 0.40	0.4666	0.33			
		FAC10.16	0	0.13	0.4898	0.31			
		Mace	2	$-$ 3.42	0.0644	1.19			
		SB062	0	$-$ 1.38	0.3183	0.50			
		SeriM82	0	5.03	0.0449	1.36			
		Suntop	0	2.95	0.2645	0.58			
		UQ114	0	$-$ 0.80	0.4347	0.36			
		Westonia	0	$-$ 0.46	0.4402	0.36			
		ZWB10.37	0	$-$ 0.32	0.4738	0.33			
		ZWW10.50	0	0.00	0.5000	0.30			

### Putative QTL for plant height

The WGNAM analysis resulted in the identification of 26 putative QTL for plant height in WAR15 and 11 in WAR16. Putative QTL for plant height with an overall or founder specific LOGP score greater than $-$ log(0.05) = 1.30 are presented in Table 7. Additionally, the effect type was identified as “overall” or “specific” depending on if the overall or founder specific LOGP score was greater than the threshold. Putative QTL were located on LGs 1A, 1B, 1D, 2A, 2B, 3A, 3B, 4B, 4D, 5B, 6A, 6B, 7A, 7B, 7D.

On LG 2B, both overall and specific effects were detected at 62.53 cM in WAR15 and 107.01 cM in WAR16. Additionally, positions LG.2B.92 in WAR15 and LG.2B.98 in WAR16 were at less than 1 cM apart in the consensus map (DArT 2018) and with a linkage disequilibrium between markers or $R^2 = 0.84$ in the MR-NAM population. QTL size for Suntop, increased the plant height by 8.19 and 6.80 cm in WAR15 and WAR16, respectively.

On LG 4D, Mace QTL size contributed negative effects for each experiment, associated with QTL at different positions (21.72 cM in WAR15 and 50.96 cM in WAR16). These QTL may be related to the Rht-D1 dwarfing allele on LG 4D that Mace carries (Table 2). Furthermore, putative QTL indicated strong allele effects from Mace in both years at different positions on LG 6A (17.57 cM in WAR16 and 42.36 cM in WAR15).

**Table 7:** Putative QTL for plant height in WAR15 and WAR16 that presented an overall or specific LOGP score greater than $-$ log(0.05) = 1.30. Putative QTL identified position, position and cloneID (DArT 2018) are shown, together with the experiment in which they were detected. For overall effects above the threshold, percentage of genetic variance attributed to the putative QTL [%var] and LOGP score are given, whereas for specific effects the founder name, size and LOGP score are provided. Effect details in bold correspond to putative QTL identified on the same linkage group for both experiments

LG	Distance (cM)	Marker	CloneID	Experiment	Effect Type	Effect Details
1A	66.23	LG.1A.48	1011620	WAR15	Overall	%var = 5.5; Score = 2.00
					Specific	Mace (Size = 7.57; Score = 2.16)
						SeriM82 (Size = − 4.46; Score = 1.33)
	105.82	LG.1A.106	2261453	WAR15	Specific	Mace (Size = 3.69; Score = 1.74)
						SB062 (Size = − 3.69; Score = 1.41)
1B	113.49	LG.1B.143	1229218	WAR15	Specific	Mace (Size = − 2.80; Score = 1.41)
1D	25.49	LG.1D.11	1099647	WAR15	Overall	%var = 2.3; Score = 1.82
					Specific	Mace (Size = − 4.90; Score = 2.82)
	58.66	LG.1D.14	3064835	WAR15	Specific	Suntop (Size = − 5.67; Score = 1.87)
	139.53	LG.1D.79	1066774	WAR15	Overall	%var = 1.8; Score = 1.90
					Specific	Mace (Size = 3.05; Score = 1.74)
						Drysdale (Size = − 3.06; Score = 1.62)
						SB062 (Size = − 2.55; Score = 1.33)
2A	75.64	LG.2A.128	2293684	WAR15	Specific	Mace (Size = − 1.82; Score = 1.40)
2B	62.53	LG.2B.92	1239537	WAR15	Overall	**%var = 13.4; Score = 2.23**
					Specific	Scout (Size = − 9.84; Score = 2.21)
						**Suntop (Size = 8.19; Score = 1.79)**
	63.52	LG.2B.98	4989040	WAR16	Specific	**Suntop (Size = 6.80; Score = 2.28)**
	107.01	LG.2B.408	1218896	WAR16	Overall	**%var = 3.6; Score = 1.31**
					Specific	SeriM82 (Size = − 4.30; Score = 1.55)
						SB062 (Size = 3.92; Score = 1.37)
3A	14.42	LG.3A.20	1056945	WAR15	Specific	SeriM82 (Size = − 4.60; Score = 1.77)
	151.45	LG.3A.193	1021077	WAR16	Overall	%var = 3; Score = 2.23
					Specific	Drysdale (Size = 5.13; Score = 2.54)
3B	55.13	LG.3B.103	1250327	WAR15	Specific	Scout (Size = − 6.43; Score = 1.62)
						Drysdale (Size = 4.33; Score = 1.3)
4B	33.74	LG.4B.27	2262825	WAR15	Specific	Mace (Size = 3.20; Score = 1.60)
4D	21.72	LG.4D.9	1201923	WAR15	Overall	%var = 14; Score = 10.66
					Specific	**Mace (Size = − 9.55; Score = 3.37)**
						Drysdale (Size = − 7.60; Score = 2.21)
						Scout (Size = 7.41; Score = 2.14)
						Dharwah dry (Size = 6.33; Score = 1.78)
	50.96	LG.4D.16	1161775	WAR16	Specific	SeriM82 (Size = 4.91; Score = 1.78)
						**Mace (Size = − 3.03; Score = 1.46)**
5B	30.46	LG.5B.89	1092379	WAR16	Specific	Suntop (Size = 2.25; Score = 2.00)
6A	17.57	LG.6A.23	1096778	WAR16	Specific	**Mace (Size = 5.43; Score = 1.47)**
	42.36	LG.6A.64	1142355	WAR15	Specific	SeriM82 (Size = 13.03; Score = 2.38)
						**Mace (Size = − 6.42; Score = 1.44)**
		LG.6A.65	1208893	WAR15	Specific	**Mace (Size = 7.50; Score = 2.03)**
						SB062 (Size = − 7.66; Score = 1.37)
	75.14	LG.6A.147	999810	WAR15	Specific	Dharwah dry (Size = 4.44; Score = 1.58)
						Mace (Size = − 2.79; Score = 1.31)
	79.64	LG.6A.154	1009888	WAR15	Specific	Suntop (Size = − 2.30; Score = 1.89)
6B	78.29	LG.6B.204	4539744	WAR16	Overall	%var = 17.6; Score = 2.74
					Specific	Suntop (Size = − 7.68; Score = 1.81)
7A	149.56	LG.7A.216	1216524	WAR16	Overall	%var = 9.5; Score = 2.11
					Specific	Drysdale (Size = − 6.72; Score = 2.05)
						Dharwah dry (Size = 5.69; Score = 1.53)
						SeriM82 (Size = 5.03; Score = 1.36)
7B	83.16	LG.7B.155	1017568	WAR15	Specific	Scout (Size = 5.13; Score = 1.61)
	104.5	LG.7B.176	1089670	WAR16	Specific	Dharwah dry (Size = 5.24; Score = 1.39)
						Mace (Size = − 5.62; Score = 1.31)
7D	80.81	LG.7D.38	1007981	WAR15	Specific	Drysdale (Size = 5.19; Score = 2.09)

Bold values correspond to Effect details

## Discussion

In this study, the powerful MPWGAIM approach described by Verbyla et al. (2014b) has been extended to NAM populations and validated on simulated data as well as on real data for a MR-NAM population, enabling a QTL analysis method that models the complexity inherent in these populations. This approach, based on a linear mixed model, consisted of a one-stage analysis of phenotypic data corresponding to individual plot data in a trial coupled with a sophisticated model for the genetic effects resulting from a genome-wide iterative search of putative QTL using molecular markers and the MR-NAM population structure.

### WGNAM performance with NAM simulated data

The simulation study showed that the WGNAM method was powerful to detect QTL, with a detection rate near 100% for QTL1 to QTL3, intermediate rates for QTL4 to QTL7 and not detecting QTL8 (Table 4). Comparing with the two methods of reference, one of the main differences was observed for QTL 4, where the effects were only present in two donors (with opposite effects). The MLMM approach could not detect the QTL but the methods WGNAM and MPP presented a detection rate of 0.646 and 0.532, respectively. This was expected given that the MLMM approach does not estimate parent specific effects while the MPP and WGNAM methods do. Another major difference occurred for QTL5 to QTL7, where WGNAM was superior with detection rates ranged from 0.378 to 0.620, MLMM detection rates were only closer to WGNAM rates for QTL6 where all donors had the same effect size, and the MPP detection rates were all under 10%. This could be due to the need to account for multiple testing in the methods of reference and Bonferroni correction being too conservative to detect QTL of smaller size.

Despite the fact that the WGNAM approach was the only method that showed a false positive rate close to the nominal type I error rate in absence of QTL effects, this result was different when the effects of eight QTL were introduced. When QTL effects were present, the mean number of false positives was 1.050, 0.714, and 0.098 for WGNAM, MLMM, and MPP methods, respectively. The WGNAM approach is more powerful, but with that power there is a modest presence of false positives. The results for the WGNAM approach are in agreement with those reported for the MPWGAIM method when three-point probabilities were implemented rather than hidden Markov model. This result was unexpected given that in the current simulation study the probabilities were calculated within the mppR package, which uses the function calc.genoprob from qtl package based on hidden Markov model.

The estimations of the QTL effects in the simulation study showed that the WGNAM approach provided greater accuracy whenever comparisons were possible to the MPP method. This result confirms the statement by (Gogel et al. 2018) that, compared to a two-stage analysis, one-stage approach ensures increased accuracy of the predicted genetic effects in a QTL mapping environment.

### Modeling the NAM population structure

The WGNAM method allows for a search of putative QTL with different effects for each of the founders in a NAM population. It does not consider just the marker scores because it may not be appropriate to assume the same QTL effect for every background present in the population. Some methodologies have incorporated this particular assumption for QTL analysis for NAM populations (Garin et al. 2017; Li et al. 2021). In fact, some similarities were found between one of the models proposed by Li et al. (2021) called *IBD.Kin-F* and the WGNAM model in “The basis of the WGNAM model” such as QTL effects based on IBD probabilities and polygenic effects. However, these methods are based on two-stage approaches that could not be followed for a partially replicated design. In this paper, the one-stage MPWGAIM method for MAGIC populations was adapted initially for a NAM population. In a NAM population a donor line is only used once, so the QTL effect is nested within the crosses between the reference and the donor lines while the reference effect is estimated across all the families. The method was extended to accommodate an MR-NAM population structure to potentially benefit from the fact that a donor can be used more than once, and so the QTL effect is estimated across the references that it was crossed to. This shows that the concept is potentially adaptable to other structured populations. Future extensions could consider that founder effects are not independent by using a relationship matrix among the founders.

Similarly to the MPWGAIM method, the linear mixed model behind the WGNAM approach considers the probability of QTL alleles inherited from the parents given their genotypes. In this study, one simple approach to estimate the probabilities was described (Table 1) which relies on founder genotypes but does not need a linkage map . However, WGNAM methodology is flexible enough to allow the user to provide the founder probabilities calculated following other procedures (Broman et al. 2003; Verbyla et al. 2014b; Li et al. 2021). This was shown in the simulation study with the implementation of IBD probabilities calculated within the mppR package.

### A whole-genome and multi-marker model

The statistical methodology behind WGNAM is equivalent to the MPWGAIM method for the QTL analysis of MAGIC population using markers positions. The base model for both methods has the capability to consider the whole-genome. That is, all molecular markers across the genome are scanned simultaneously, rather than each marker separately. A key component to achieve this is the dimension reduction in a large data problem to the number of lines ${n_g}$ which can be much smaller than the effective dimension in multi-parent situations (different effects for each founder at each locus, dimension $\ge {n_f} \times {n_m}$). The dimensionality reduction works together with the strategy to test the presence of QTL and identify the position using the outlier statistic. As a result, the QTL search performed by WGNAM avoids both repeated scans for every locus and the requirement for correction due to multiple testing. The results for the simulation study showed this strategy keeps the false positive rate, as per the probability that at least one QTL found when none exist, at the set threshold, agreeing with previous simulation studies (Verbyla et al. 2007, 2014b).

Furthermore, an iterative process based on forward variable selection is carried out to find additional QTL. Given a significance level, at each iteration, a likelihood ratio test is performed to support the search for additional QTL. Some methodologies carry out a multi-QTL detection model (Verbyla et al. 2007; Segura et al. 2012; Verbyla et al. 2014b) but none of them was developed for NAM populations. In NAM populations, the MPP method (Garin et al. 2017) tests each single marker at a time and builds a list of detected QTL based on a threshold, that subset of QTL is then used to build the multi-QTL model. Compared to single marker approaches, the WGNAM requires only a small number of iterations to build an exhaustive multi-marker model, providing a final linear mixed model for a MR-NAM population. Furthermore, compared to the multi-QTL approaches, the simulation study showed that the WGNAM methodology is more powerful, but with that power there is a modest presence of false positives.

### One-stage approach

The proposed methodology is based on a one-stage analysis where non-genetic effects, such as terms for the experimental design, are easily included in the model. This was crucial in the analysis of plant height data for the wheat MR-NAM population where a partially replicated experimental design was followed and spatial effects were significant in both years of experiments. Given the size of NAM populations, the presence of spatial effects is unavoidable and having a good experimental design as well as the ability to model the spatial effects becomes essential for sound phenotypic assessment. Despite being computationally demanding compared to a two-stage analysis, this one-stage approach ensured increased accuracy of the predicted genetic effects in the simulation study, which is of particular interest for traits with low heritability.

### Plant height QTL for wheat

The WGNAM analysis was demonstrated using a wheat MR-NAM population in order to perform QTL mapping for plant height measured at two different field trials. A total of 37 putative QTL were detected in this study, 26 were detected in the WAR15 experiment and 11 in the WAR16 experiment. Genetic studies in wheat have identified 25 reduced height (Rht) genes and major QTL for plant height on almost all LGs (except for 1D, 3D, 6B, and 7D) according to Komugi wheat gene catalog (Komugi, n.d.). Most current wheat lines contain Rht-B1b (formerly Rht1) or Rht-D1b (formerly Rht2) to reduce plant height and increase grain yield (Ellis et al. 2005).

One of the major-effect QTL detected in this study (LOGP score = 10.66, Table 7) is mapped on LG 4D at 21.72 cM according to the wheat consensus map v4.0 (DArT 2018). This is probably related to the Green Revolution height-reducing gene Rht-D1 mapped on LG 4D at 20.07 cM in the consensus map (named RHTD1 in DArT (2018)). Furthermore, Mace and Drysdale showed height reducing allele effects (Table 7) in agreement with them carrying the Rht-D1b dwarfing allele (Table 2). No significant evidence was observed for Westonia that also carries the Rht1-D1b allele but this may be explained by the fact that Westonia was only crossed to Mace and no segregation was expected for this gene in that family.

Another major dwarfing gene Rht-B1 is known to be located on the short arm of LG 4B. Both years of trials showed evidence of a putative QTL in that LG at 33.74 cM in WAR15 (Table 7) and 33.55 cM in WAR16 (Supplementary Table S1). In both years alleles coming from Mace increased plant height (in WAR15 LOGP score = 1.60 Table 7, in WAR16 LOGP score = 1.19 Supplementary Table S1). Rht-B1 does not appear on the consensus map (DArT 2018), but both QTL are likely to coincide with the major dwarfing gene Rht-B1 (Richard 2017; Christopher et al. 2021). Mace was one of the only reference parents lacking the Rht-B1b allele (Table 2). Drysdale also does not carry that allele but, as a donor, its allele was potentially present in fewer MR-NAM lines, so the likelihood for detecting a significant effect might have been affected (Drysdale had a plant height increasing effect in WAR16 with a LOGP score = 1.18). Another limitation to the detection of plant height QTL could be the moderate selection pressure against plant height variability carried out during the development of this MR-NAM.

Collocation of QTL between experiments was only observed on LG 2B (Table 7). In both experiments, the Suntop allele increased plant height. This could be collocated with Rht-4 also mapped on that LG. Possible reasons for not finding more collocated QTL across experiments include the interaction of genotype by environment for plant height, the lack of coverage of some LG regions, and also that not all the MR-NAM lines were tested in the second year of trials (Table 2). The reduction in MR-NAM population size may have affected the diversity of alleles as well as their frequency in the population. Further efforts are needed to extend the current model to deal with NAM populations at multiple environments that incorporate the interaction with the environment (Verbyla et al. 2014a).

The WGNAM method provided scores to measure the strength of the putative QTL either for specific parents or for the overall MR-NAM population. Additionally, it offered estimates of plant height founder specific putative QTL sizes (Table 7). This information can facilitate the identification of the parental origin of favorable alleles at each QTL which can be appealing for line selection purposes given that the founders were commercial lines.

The use of the WGNAM method to perform an association study for plant height in an MR-NAM population demonstrated that WGNAM can be implemented to explore other traits of interest to provide insights into genetic mechanisms in wheat which will allow breeders to deliver commercial lines to growers with improved characteristics (Christopher et al. 2021).

### Conclusion

A method to perform QTL analysis in NAM or MR-NAM populations has been adapted from the already demonstrated powerful methodology in MAGIC populations (Verbyla et al. 2014b). The WGNAM method was assessed and compared to other methods through simulated data and applied to a wheat MR-NAM population for plant height in two environments. Following the same strategy of MPWGAIM, WGNAM results in a powerful QTL detection that makes use of the population structure to keep the benefits of multi-parent populations while offering, at the same time, an understanding of the QTL effects. This extension of MPWGAIM originally designed for MAGIC demonstrates the potential for it to perform QTL mapping studies in other multi-parent populations structures.

## Supplementary Information

Below is the link to the electronic supplementary material.

Supplementary file 1 (PDF 535 kb)
