# Supplementary material for: WGNAM: whole-genome nested association mapping
Source: Theor Appl Genet. 2022 May 21;135(7):2213–32. doi: 10.1007/s00122-022-04107-x (PMC9271119; doi:10.1007/s00122-022-04107-x)
Supplement: Supplementary file 1 — (PDF 535 kb) [file 122_2022_4107_MOESM1_ESM.pdf]

Theoretical and Applied Genetics

WGNAM: whole-genome nested association mapping

Maria Valeria Paccapelo[1], Alison M. Kelly[1], Jack T. Christopher[2], and Arūnas P. Verbyla[3]

[1] Department of Agriculture and Fisheries, Leslie Research Facility, Toowoomba, QLD 4350, Australia

[2] The University of Queensland, Queensland Alliance for Agriculture and Food Innovation (QAAFI), Leslie Research Facility, Toowoomba, QLD 4350, Australia

[3] Data61, CSIRO, Atherton, QLD 4883, Australia

Corresponding author: Maria Valeria Paccapelo (E-mail: Valeria.Paccapelo@daf.qld.gov.au)

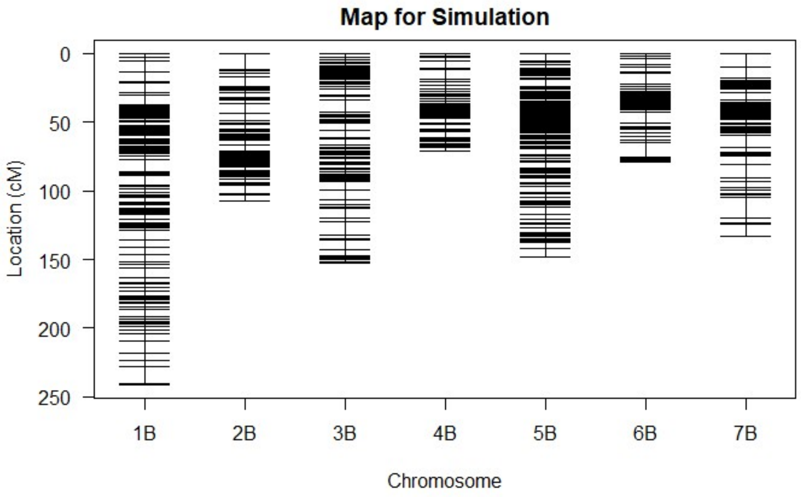

Supplementary Figure 1: Subset of the DArT consensus map used for the simulated data

Theoretical and Applied Genetics

WGNAM: whole-genome nested association mapping

Maria Valeria Paccapelo[1], Alison M. Kelly[1], Jack T. Christopher[2], and Arūnas P. Verbyla[3]

[1] Department of Agriculture and Fisheries, Leslie Research Facility, Toowoomba, QLD 4350, Australia

[2] The University of Queensland, Queensland Alliance for Agriculture and Food Innovation (QAAFI), Leslie Research Facility, Toowoomba, QLD 4350, Australia

[3] Data61, CSIRO, Atherton, QLD 4883, Australia

Corresponding author: Maria Valeria Paccapelo (E-mail: Valeria.Paccapelo@daf.qld.gov.au)

Supplementary Table 1: Complete list of putative QTL for plant height in WAR15 and WAR16. For each experiment, putative QTL identified location, position and cloneID (in consensus map DArT, 2018) are shown. For overall effects, percentage of genetic variance attributed to the putative QTL [%var] and LOGP score are given whereas for specific effects the founder name, size and LOGP score are provided.

								Founder Specific			Overall		
Order	Experiment	Iteration	LG	Distance (cM)	Marker	CloneID	Founder	Size	Probability	Score	Probability2	% var	Score2
1	WAR15	2	1A	66.23	LG.1A.48	1011620	Dharwah dry	0.751	0.438	0.36	0.01	5.5	2
2	WAR15	2	1A	66.23	LG.1A.48	1011620	Drysdale	-0.513	0.457	0.34			
3	WAR15	2	1A	66.23	LG.1A.48	1011620	EGA Gregory	-0.167	0.486	0.31			
4	WAR15	2	1A	66.23	LG.1A.48	1011620	EGA Wylie	-0.28	0.477	0.32			
5	WAR15	2	1A	66.23	LG.1A.48	1011620	FAC10.16	0.016	0.499	0.3			
6	WAR15	2	1A	66.23	LG.1A.48	1011620	Mace	7.572	0.007	2.16			
7	WAR15	2	1A	66.23	LG.1A.48	1011620	UQ114	0.096	0.492	0.31			
8	WAR15	2	1A	66.23	LG.1A.48	1011620	SB062	1.104	0.41	0.39			
9	WAR15	2	1A	66.23	LG.1A.48	1011620	Scout	-2.782	0.182	0.74			
10	WAR15	2	1A	66.23	LG.1A.48	1011620	SeriM82	-4.455	0.047	1.33			
11	WAR15	2	1A	66.23	LG.1A.48	1011620	Suntop	-1.155	0.35	0.46			
12	WAR15	2	1A	66.23	LG.1A.48	1011620	Westonia	-0.242	0.48	0.32			
13	WAR15	2	1A	66.23	LG.1A.48	1011620	ZWB10.37	-0.402	0.464	0.33			
14	WAR15	2	1A	66.23	LG.1A.48	1011620	ZWW10.50	0.455	0.462	0.33			
15	WAR15	24	1A	105.82	LG.1A.106	2261453	Dharwah dry	-0.138	0.474	0.32	0.104	2	0.98
16	WAR15	24	1A	105.82	LG.1A.106	2261453	Drysdale	-0.926	0.306	0.51			
17	WAR15	24	1A	105.82	LG.1A.106	2261453	EGA Gregory	-0.05	0.493	0.31			
18	WAR15	24	1A	105.82	LG.1A.106	2261453	EGA Wylie	0.661	0.377	0.42			
19	WAR15	24	1A	105.82	LG.1A.106	2261453	FAC10.16	-0.059	0.488	0.31			
20	WAR15	24	1A	105.82	LG.1A.106	2261453	Mace	3.691	0.018	1.74			
21	WAR15	24	1A	105.82	LG.1A.106	2261453	UQ114	1.175	0.258	0.59			
22	WAR15	24	1A	105.82	LG.1A.106	2261453	SB062	-3.692	0.039	1.41			
23	WAR15	24	1A	105.82	LG.1A.106	2261453	Scout	2.793	0.089	1.05			
24	WAR15	24	1A	105.82	LG.1A.106	2261453	SeriM82	-1.627	0.21	0.68			
25	WAR15	24	1A	105.82	LG.1A.106	2261453	Suntop	-0.987	0.247	0.61			
26	WAR15	24	1A	105.82	LG.1A.106	2261453	Westonia	0.197	0.466	0.33			
27	WAR15	24	1A	105.82	LG.1A.106	2261453	ZWB10.37	-1.174	0.317	0.5			
28	WAR15	24	1A	105.82	LG.1A.106	2261453	ZWW10.50	0.137	0.48	0.32			
29	WAR15	26	1B	113.49	LG.1B.143	1229218	Dharwah dry	-0.054	0.49	0.31	0.336	2.5	0.47
30	WAR15	26	1B	113.49	LG.1B.143	1229218	Drysdale	0.08	0.484	0.31			
31	WAR15	26	1B	113.49	LG.1B.143	1229218	EGA Gregory	-0.056	0.492	0.31			
32	WAR15	26	1B	113.49	LG.1B.143	1229218	EGA Wylie	-0.094	0.487	0.31			
33	WAR15	26	1B	113.49	LG.1B.143	1229218	FAC10.16	0.005	0.499	0.3			
34	WAR15	26	1B	113.49	LG.1B.143	1229218	Mace	-2.795	0.039	1.41			
35	WAR15	26	1B	113.49	LG.1B.143	1229218	UQ114	0.032	0.496	0.3			
36	WAR15	26	1B	113.49	LG.1B.143	1229218	SB062	2.93	0.081	1.09			
37	WAR15	26	1B	113.49	LG.1B.143	1229218	Scout	-2.966	0.1	1			
38	WAR15	26	1B	113.49	LG.1B.143	1229218	SeriM82	0.473	0.413	0.38			
39	WAR15	26	1B	113.49	LG.1B.143	1229218	Suntop	3.189	0.075	1.12			
40	WAR15	26	1B	113.49	LG.1B.143	1229218	Westonia	-0.153	0.474	0.32			
41	WAR15	26	1B	113.49	LG.1B.143	1229218	ZWB10.37	-0.037	0.495	0.31			
42	WAR15	26	1B	113.49	LG.1B.143	1229218	ZWW10.50	-0.556	0.402	0.4			
43	WAR15	1	1D	25.49	LG.1D.11	1099647	Dharwah dry	-1.966	0.201	0.7	0.015	2.3	1.82
44	WAR15	1	1D	25.49	LG.1D.11	1099647	Drysdale	-1.551	0.263	0.58			
45	WAR15	1	1D	25.49	LG.1D.11	1099647	EGA Gregory	-0.081	0.491	0.31			
46	WAR15	1	1D	25.49	LG.1D.11	1099647	EGA Wylie	-0.136	0.484	0.31			
47	WAR15	1	1D	25.49	LG.1D.11	1099647	FAC10.16	0.008	0.499	0.3			
48	WAR15	1	1D	25.49	LG.1D.11	1099647	Mace	-4.903	0.002	2.82			
49	WAR15	1	1D	25.49	LG.1D.11	1099647	UQ114	0.047	0.495	0.31			
50	WAR15	1	1D	25.49	LG.1D.11	1099647	SB062	1.05	0.329	0.48			
51	WAR15	1	1D	25.49	LG.1D.11	1099647	Scout	0.58	0.433	0.36			
52	WAR15	1	1D	25.49	LG.1D.11	1099647	SeriM82	3.138	0.101	1			
53	WAR15	1	1D	25.49	LG.1D.11	1099647	Suntop	0.11	0.487	0.31			
54	WAR15	1	1D	25.49	LG.1D.11	1099647	Westonia	1.109	0.333	0.48			
55	WAR15	1	1D	25.49	LG.1D.11	1099647	ZWB10.37	-0.054	0.494	0.31			
56	WAR15	1	1D	25.49	LG.1D.11	1099647	ZWW10.50	2.649	0.149	0.83			
57	WAR15	19	1D	58.66	LG.1D.14	3064835	Dharwah dry	0.443	0.453	0.34	0.302	4.2	0.52
58	WAR15	19	1D	58.66	LG.1D.14	3064835	Drysdale	2.047	0.166	0.78			
59	WAR15	19	1D	58.66	LG.1D.14	3064835	EGA Gregory	-0.098	0.49	0.31			
60	WAR15	19	1D	58.66	LG.1D.14	3064835	EGA Wylie	-0.165	0.483	0.32			
61	WAR15	19	1D	58.66	LG.1D.14	3064835	FAC10.16	0.009	0.499	0.3			
62	WAR15	19	1D	58.66	LG.1D.14	3064835	Mace	0.124	0.477	0.32			
63	WAR15	19	1D	58.66	LG.1D.14	3064835	UQ114	0.057	0.494	0.31			
64	WAR15	19	1D	58.66	LG.1D.14	3064835	SB062	0.65	0.432	0.36			
65	WAR15	19	1D	58.66	LG.1D.14	3064835	Scout	3.572	0.088	1.06			
66	WAR15	19	1D	58.66	LG.1D.14	3064835	SeriM82	-0.654	0.431	0.37			
67	WAR15	19	1D	58.66	LG.1D.14	3064835	Suntop	-5.671	0.013	1.87			
68	WAR15	19	1D	58.66	LG.1D.14	3064835	Westonia	-1.865	0.249	0.6			
69	WAR15	19	1D	58.66	LG.1D.14	3064835	ZWB10.37	-0.065	0.493	0.31			

								Founder Specific			Overall		
Order	Experiment	Iteration	LG	Distance (cM)	Marker	CloneID	Founder	Size	Probability	Score	Probability2	% var	Score2
70	WAR15	19	1D	58.66	LG.1D.14	3064835	ZWW10.50	1.616	0.282	0.55			
71	WAR15	14	1D	139.53	LG.1D.79	1066774	Dharwah dry	0.615	0.341	0.47	0.013	1.8	1.9
72	WAR15	14	1D	139.53	LG.1D.79	1066774	Drysdale	-3.058	0.024	1.62			
73	WAR15	14	1D	139.53	LG.1D.79	1066774	EGA Gregory	-0.045	0.493	0.31			
74	WAR15	14	1D	139.53	LG.1D.79	1066774	EGA Wylie	-1.741	0.21	0.68			
75	WAR15	14	1D	139.53	LG.1D.79	1066774	FAC10.16	0.004	0.499	0.3			
76	WAR15	14	1D	139.53	LG.1D.79	1066774	Mace	3.052	0.018	1.74			
77	WAR15	14	1D	139.53	LG.1D.79	1066774	UQ114	0.026	0.496	0.3			
78	WAR15	14	1D	139.53	LG.1D.79	1066774	SB062	-2.552	0.047	1.33			
79	WAR15	14	1D	139.53	LG.1D.79	1066774	Scout	-1.055	0.227	0.64			
80	WAR15	14	1D	139.53	LG.1D.79	1066774	SeriM82	0.522	0.363	0.44			
81	WAR15	14	1D	139.53	LG.1D.79	1066774	Suntop	2.025	0.057	1.25			
82	WAR15	14	1D	139.53	LG.1D.79	1066774	Westonia	-0.066	0.49	0.31			
83	WAR15	14	1D	139.53	LG.1D.79	1066774	ZWB10.37	2.148	0.148	0.83			
84	WAR15	14	1D	139.53	LG.1D.79	1066774	ZWW10.50	0.123	0.481	0.32			
85	WAR15	22	2A	75.64	LG.2A.128	2293684	Dharwah dry	-0.22	0.432	0.36	0.24	0.7	0.62
86	WAR15	22	2A	75.64	LG.2A.128	2293684	Drysdale	1.722	0.088	1.06			
87	WAR15	22	2A	75.64	LG.2A.128	2293684	EGA Gregory	-0.409	0.39	0.41			
88	WAR15	22	2A	75.64	LG.2A.128	2293684	EGA Wylie	-0.394	0.396	0.4			
89	WAR15	22	2A	75.64	LG.2A.128	2293684	FAC10.16	-0.51	0.363	0.44			
90	WAR15	22	2A	75.64	LG.2A.128	2293684	Mace	-1.816	0.04	1.4			
91	WAR15	22	2A	75.64	LG.2A.128	2293684	UQ114	-0.618	0.335	0.47			
92	WAR15	22	2A	75.64	LG.2A.128	2293684	SB062	1.104	0.189	0.72			
93	WAR15	22	2A	75.64	LG.2A.128	2293684	Scout	0.138	0.468	0.33			
94	WAR15	22	2A	75.64	LG.2A.128	2293684	SeriM82	1.811	0.08	1.1			
95	WAR15	22	2A	75.64	LG.2A.128	2293684	Suntop	-1.394	0.06	1.22			
96	WAR15	22	2A	75.64	LG.2A.128	2293684	Westonia	0.041	0.489	0.31			
97	WAR15	22	2A	75.64	LG.2A.128	2293684	ZWB10.37	-0.226	0.439	0.36			
98	WAR15	22	2A	75.64	LG.2A.128	2293684	ZWW10.50	0.771	0.305	0.52			
113	WAR15	18	2B	62.53	LG.2B.92	1239537	Dharwah dry	1.355	0.414	0.38	0.006	13.4	2.23
114	WAR15	18	2B	62.53	LG.2B.92	1239537	Drysdale	2.072	0.277	0.56			
115	WAR15	18	2B	62.53	LG.2B.92	1239537	EGA Gregory	-0.301	0.481	0.32			
116	WAR15	18	2B	62.53	LG.2B.92	1239537	EGA Wylie	-0.505	0.469	0.33			
117	WAR15	18	2B	62.53	LG.2B.92	1239537	FAC10.16	0.029	0.498	0.3			
118	WAR15	18	2B	62.53	LG.2B.92	1239537	Mace	-1.345	0.367	0.44			
119	WAR15	18	2B	62.53	LG.2B.92	1239537	UQ114	0.173	0.489	0.31			
120	WAR15	18	2B	62.53	LG.2B.92	1239537	SB062	1.991	0.375	0.43			
121	WAR15	18	2B	62.53	LG.2B.92	1239537	Scout	-9.84	0.006	2.21			
122	WAR15	18	2B	62.53	LG.2B.92	1239537	SeriM82	-2.002	0.374	0.43			
123	WAR15	18	2B	62.53	LG.2B.92	1239537	Suntop	8.189	0.016	1.79			
124	WAR15	18	2B	62.53	LG.2B.92	1239537	Westonia	-0.437	0.472	0.33			
125	WAR15	18	2B	62.53	LG.2B.92	1239537	ZWB10.37	-0.2	0.488	0.31			
126	WAR15	18	2B	62.53	LG.2B.92	1239537	ZWW10.50	0.821	0.448	0.35			
155	WAR15	20	3A	14.42	LG.3A.20	1056945	Dharwah dry	0.412	0.455	0.34	0.594	1.7	0.23
156	WAR15	20	3A	14.42	LG.3A.20	1056945	Drysdale	-0.281	0.469	0.33			
157	WAR15	20	3A	14.42	LG.3A.20	1056945	EGA Gregory	-0.091	0.49	0.31			
158	WAR15	20	3A	14.42	LG.3A.20	1056945	EGA Wylie	-0.153	0.483	0.32			
159	WAR15	20	3A	14.42	LG.3A.20	1056945	FAC10.16	0.009	0.499	0.3			
160	WAR15	20	3A	14.42	LG.3A.20	1056945	Mace	-0.57	0.413	0.38			
161	WAR15	20	3A	14.42	LG.3A.20	1056945	UQ114	0.053	0.494	0.31			
162	WAR15	20	3A	14.42	LG.3A.20	1056945	SB062	0.605	0.434	0.36			
163	WAR15	20	3A	14.42	LG.3A.20	1056945	Scout	-0.962	0.376	0.42			
164	WAR15	20	3A	14.42	LG.3A.20	1056945	SeriM82	-4.599	0.017	1.77			
165	WAR15	20	3A	14.42	LG.3A.20	1056945	Suntop	2.668	0.147	0.83			
166	WAR15	20	3A	14.42	LG.3A.20	1056945	Westonia	-0.133	0.486	0.31			
167	WAR15	20	3A	14.42	LG.3A.20	1056945	ZWB10.37	2.796	0.177	0.75			
168	WAR15	20	3A	14.42	LG.3A.20	1056945	ZWW10.50	0.249	0.473	0.33			
183	WAR15	10	3B	55.13	LG.3B.103	1250327	Dharwah dry	0.655	0.443	0.35	0.261	3.6	0.58
184	WAR15	10	3B	55.13	LG.3B.103	1250327	Drysdale	4.328	0.05	1.3			
185	WAR15	10	3B	55.13	LG.3B.103	1250327	EGA Gregory	-0.145	0.487	0.31			
186	WAR15	10	3B	55.13	LG.3B.103	1250327	EGA Wylie	-0.244	0.479	0.32			
187	WAR15	10	3B	55.13	LG.3B.103	1250327	FAC10.16	0.014	0.499	0.3			
188	WAR15	10	3B	55.13	LG.3B.103	1250327	Mace	1.359	0.334	0.48			
189	WAR15	10	3B	55.13	LG.3B.103	1250327	UQ114	0.084	0.493	0.31			
190	WAR15	10	3B	55.13	LG.3B.103	1250327	SB062	0.962	0.416	0.38			
191	WAR15	10	3B	55.13	LG.3B.103	1250327	Scout	-6.43	0.024	1.62			
192	WAR15	10	3B	55.13	LG.3B.103	1250327	SeriM82	-0.968	0.415	0.38			
193	WAR15	10	3B	55.13	LG.3B.103	1250327	Suntop	0.297	0.461	0.34			
194	WAR15	10	3B	55.13	LG.3B.103	1250327	Westonia	-0.211	0.482	0.32			
195	WAR15	10	3B	55.13	LG.3B.103	1250327	ZWB10.37	-0.097	0.492	0.31			
196	WAR15	10	3B	55.13	LG.3B.103	1250327	ZWW10.50	0.397	0.465	0.33			
211	WAR15	15	4B	33.74	LG.4B.27	2262825	Dharwah dry	-1.318	0.267	0.57	0.488	1.2	0.31
212	WAR15	15	4B	33.74	LG.4B.27	2262825	Drysdale	0.797	0.344	0.46			
213	WAR15	15	4B	33.74	LG.4B.27	2262825	EGA Gregory	-0.051	0.493	0.31			
214	WAR15	15	4B	33.74	LG.4B.27	2262825	EGA Wylie	-0.086	0.488	0.31			
215	WAR15	15	4B	33.74	LG.4B.27	2262825	FAC10.16	0.005	0.499	0.3			
216	WAR15	15	4B	33.74	LG.4B.27	2262825	Mace	3.2	0.025	1.6			
217	WAR15	15	4B	33.74	LG.4B.27	2262825	UQ114	0.03	0.496	0.3			
218	WAR15	15	4B	33.74	LG.4B.27	2262825	SB062	-1.61	0.219	0.66			
219	WAR15	15	4B	33.74	LG.4B.27	2262825	Scout	-1.334	0.276	0.56			
220	WAR15	15	4B	33.74	LG.4B.27	2262825	SeriM82	2.125	0.158	0.8			

								Founder Specific			Overall		
Order	Experiment	Iteration	LG	Distance (cM)	Marker	CloneID	Founder	Size	Probability	Score	Probability2	% var	Score2
221	WAR15	15	4B	33.74	LG.4B.27	2262825	Suntop	0.815	0.353	0.45			
222	WAR15	15	4B	33.74	LG.4B.27	2262825	Westonia	-0.074	0.489	0.31			
223	WAR15	15	4B	33.74	LG.4B.27	2262825	ZWB10.37	-0.034	0.495	0.31			
224	WAR15	15	4B	33.74	LG.4B.27	2262825	ZWW10.50	-2.464	0.143	0.84			
225	WAR15	5	4D	21.72	LG.4D.9	1201923	Dharwah dry	6.333	0.017	1.78	0	14	10.66
226	WAR15	5	4D	21.72	LG.4D.9	1201923	Drysdale	-7.602	0.006	2.21			
227	WAR15	5	4D	21.72	LG.4D.9	1201923	EGA Gregory	0.866	0.393	0.41			
228	WAR15	5	4D	21.72	LG.4D.9	1201923	EGA Wylie	-2.764	0.198	0.7			
229	WAR15	5	4D	21.72	LG.4D.9	1201923	FAC10.16	0.021	0.498	0.3			
230	WAR15	5	4D	21.72	LG.4D.9	1201923	Mace	-9.553	0	3.37			
231	WAR15	5	4D	21.72	LG.4D.9	1201923	UQ114	1.487	0.317	0.5			
232	WAR15	5	4D	21.72	LG.4D.9	1201923	SB062	-1.067	0.361	0.44			
233	WAR15	5	4D	21.72	LG.4D.9	1201923	Scout	7.406	0.007	2.14			
234	WAR15	5	4D	21.72	LG.4D.9	1201923	SeriM82	-0.174	0.477	0.32			
235	WAR15	5	4D	21.72	LG.4D.9	1201923	Suntop	1.481	0.276	0.56			
236	WAR15	5	4D	21.72	LG.4D.9	1201923	Westonia	-0.321	0.477	0.32			
237	WAR15	5	4D	21.72	LG.4D.9	1201923	ZWB10.37	-0.147	0.489	0.31			
238	WAR15	5	4D	21.72	LG.4D.9	1201923	ZWW10.50	4.035	0.133	0.88			
253	WAR15	17	6A	42.36	LG.6A.64	1142355	Dharwah dry	-1.119	0.41	0.39	0.057	12	1.25
254	WAR15	17	6A	42.36	LG.6A.64	1142355	Drysdale	**-5.683**	**0.103**	0.99			
255	WAR15	17	6A	42.36	LG.6A.64	1142355	EGA Gregory	-0.39	0.478	0.32			
256	WAR15	17	6A	42.36	LG.6A.64	1142355	EGA Wylie	-0.656	0.464	0.33			
257	WAR15	17	6A	42.36	LG.6A.64	1142355	FAC10.16	0.037	0.498	0.3			
258	WAR15	17	6A	42.36	LG.6A.64	1142355	Mace	**-6.415**	**0.036**	1.44			
259	WAR15	17	6A	42.36	LG.6A.64	1142355	UQ114	0.225	0.487	0.31			
260	WAR15	17	6A	42.36	LG.6A.64	1142355	SB062	-1.742	0.356	0.45			
261	WAR15	17	6A	42.36	LG.6A.64	1142355	Scout	2.796	0.346	0.46			
262	WAR15	17	6A	42.36	LG.6A.64	1142355	SeriM82	**13.032**	**0.004**	2.38			
263	WAR15	17	6A	42.36	LG.6A.64	1142355	Suntop	0.53	0.47	0.33			
264	WAR15	17	6A	42.36	LG.6A.64	1142355	Westonia	**-5.809**	**0.142**	0.85			
265	WAR15	17	6A	42.36	LG.6A.64	1142355	ZWB10.37	-0.26	0.486	0.31			
266	WAR15	17	6A	42.36	LG.6A.64	1142355	ZWW10.50	**5.453**	**0.123**	0.91			
267	WAR15	13	6A	42.36	LG.6A.65	1208893	Dharwah dry	**-5.314**	**0.125**	0.9	0.144	8.6	0.84
268	WAR15	13	6A	42.36	LG.6A.65	1208893	Drysdale	**5.303**	**0.102**	0.99			
269	WAR15	13	6A	42.36	LG.6A.65	1208893	EGA Gregory	-0.271	0.482	0.32			
270	WAR15	13	6A	42.36	LG.6A.65	1208893	EGA Wylie	-0.455	0.47	0.33			
271	WAR15	13	6A	42.36	LG.6A.65	1208893	FAC10.16	0.026	0.498	0.3			
272	WAR15	13	6A	42.36	LG.6A.65	1208893	Mace	**7.495**	**0.009**	2.03			
273	WAR15	13	6A	42.36	LG.6A.65	1208893	UQ114	0.156	0.49	0.31			
274	WAR15	13	6A	42.36	LG.6A.65	1208893	SB062	**-7.66**	**0.043**	1.37			
275	WAR15	13	6A	42.36	LG.6A.65	1208893	Scout	1.941	0.374	0.43			
276	WAR15	13	6A	42.36	LG.6A.65	1208893	SeriM82	-2.109	0.323	0.49			
277	WAR15	13	6A	42.36	LG.6A.65	1208893	Suntop	0.368	0.476	0.32			
278	WAR15	13	6A	42.36	LG.6A.65	1208893	Westonia	-1.212	0.403	0.39			
279	WAR15	13	6A	42.36	LG.6A.65	1208893	ZWB10.37	-0.181	0.488	0.31			
280	WAR15	13	6A	42.36	LG.6A.65	1208893	ZWW10.50	1.913	0.327	0.49			
281	WAR15	12	6A	75.14	LG.6A.147	999810	Dharwah dry	4.444	0.026	1.58	0.5	1.6	0.3
282	WAR15	12	6A	75.14	LG.6A.147	999810	Drysdale	-2.369	0.16	0.79			
283	WAR15	12	6A	75.14	LG.6A.147	999810	EGA Gregory	-0.068	0.491	0.31			
284	WAR15	12	6A	75.14	LG.6A.147	999810	EGA Wylie	-0.115	0.486	0.31			
285	WAR15	12	6A	75.14	LG.6A.147	999810	FAC10.16	0.007	0.499	0.3			
286	WAR15	12	6A	75.14	LG.6A.147	999810	Mace	-2.793	0.049	1.31			
287	WAR15	12	6A	75.14	LG.6A.147	999810	UQ114	0.04	0.495	0.31			
288	WAR15	12	6A	75.14	LG.6A.147	999810	SB062	0.13	0.478	0.32			
289	WAR15	12	6A	75.14	LG.6A.147	999810	Scout	0.49	0.439	0.36			
290	WAR15	12	6A	75.14	LG.6A.147	999810	SeriM82	-0.605	0.409	0.39			
291	WAR15	12	6A	75.14	LG.6A.147	999810	Suntop	0.093	0.488	0.31			
292	WAR15	12	6A	75.14	LG.6A.147	999810	Westonia	0.722	0.396	0.4			
293	WAR15	12	6A	75.14	LG.6A.147	999810	ZWB10.37	-0.046	0.494	0.31			
294	WAR15	12	6A	75.14	LG.6A.147	999810	ZWW10.50	0.072	0.489	0.31			
295	WAR15	21	6A	79.64	LG.6A.154	1009888	Dharwah dry	-0.736	0.313	0.5	0.59	0.7	0.23
296	WAR15	21	6A	79.64	LG.6A.154	1009888	Drysdale	1.118	0.233	0.63			
297	WAR15	21	6A	79.64	LG.6A.154	1009888	EGA Gregory	0.453	0.385	0.41			
298	WAR15	21	6A	79.64	LG.6A.154	1009888	EGA Wylie	-0.037	0.492	0.31			
299	WAR15	21	6A	79.64	LG.6A.154	1009888	FAC10.16	1.087	0.239	0.62			
300	WAR15	21	6A	79.64	LG.6A.154	1009888	Mace	-0.19	0.459	0.34			
301	WAR15	21	6A	79.64	LG.6A.154	1009888	UQ114	-1.033	0.257	0.59			
302	WAR15	21	6A	79.64	LG.6A.154	1009888	SB062	0.656	0.33	0.48			
303	WAR15	21	6A	79.64	LG.6A.154	1009888	Scout	1.205	0.223	0.65			
304	WAR15	21	6A	79.64	LG.6A.154	1009888	SeriM82	0.248	0.435	0.36			
305	WAR15	21	6A	79.64	LG.6A.154	1009888	Suntop	-2.297	0.013	1.89			
306	WAR15	21	6A	79.64	LG.6A.154	1009888	Westonia	-0.032	0.493	0.31			
307	WAR15	21	6A	79.64	LG.6A.154	1009888	ZWB10.37	-0.502	0.374	0.43			
308	WAR15	21	6A	79.64	LG.6A.154	1009888	ZWW10.50	0.061	0.487	0.31			
337	WAR15	23	7B	83.16	LG.7B.155	1017568	Dharwah dry	0.43	0.454	0.34	0.547	2.3	0.26
338	WAR15	23	7B	83.16	LG.7B.155	1017568	Drysdale	-2.681	0.109	0.96			
339	WAR15	23	7B	83.16	LG.7B.155	1017568	EGA Gregory	-0.095	0.49	0.31			
340	WAR15	23	7B	83.16	LG.7B.155	1017568	EGA Wylie	-0.16	0.483	0.32			
341	WAR15	23	7B	83.16	LG.7B.155	1017568	FAC10.16	0.009	0.499	0.3			
342	WAR15	23	7B	83.16	LG.7B.155	1017568	Mace	-1.814	0.242	0.62			
343	WAR15	23	7B	83.16	LG.7B.155	1017568	UQ114	0.055	0.494	0.31			

								Founder Specific			Overall		
Order	Experiment	Iteration	LG	Distance (cM)	Marker	CloneID	Founder	Size	Probability	Score	Probability2	% var	Score2
344	WAR15	23	7B	83.16	LG.7B.155	1017568	SB062	0.631	0.433	0.36			
345	WAR15	23	7B	83.16	LG.7B.155	1017568	Scout	5.132	0.025	1.61			
346	WAR15	23	7B	83.16	LG.7B.155	1017568	SeriM82	-0.635	0.432	0.36			
347	WAR15	23	7B	83.16	LG.7B.155	1017568	Suntop	-0.93	0.359	0.45			
348	WAR15	23	7B	83.16	LG.7B.155	1017568	Westonia	-0.138	0.485	0.31			
349	WAR15	23	7B	83.16	LG.7B.155	1017568	ZWB10.37	-0.064	0.493	0.31			
350	WAR15	23	7B	83.16	LG.7B.155	1017568	ZWW10.50	0.26	0.472	0.33			
351	WAR15	9	7D	80.81	LG.7D.38	1007981	Dharwah dry	0.485	0.451	0.35	0.084	3	1.07
352	WAR15	9	7D	80.81	LG.7D.38	1007981	Drysdale	5.188	0.008	2.09			
353	WAR15	9	7D	80.81	LG.7D.38	1007981	EGA Gregory	-0.108	0.489	0.31			
354	WAR15	9	7D	80.81	LG.7D.38	1007981	EGA Wylie	-0.181	0.482	0.32			
355	WAR15	9	7D	80.81	LG.7D.38	1007981	FAC10.16	0.01	0.499	0.3			
356	WAR15	9	7D	80.81	LG.7D.38	1007981	Mace	2.888	0.135	0.87			
357	WAR15	9	7D	80.81	LG.7D.38	1007981	UQ114	-3.288	0.078	1.11			
358	WAR15	9	7D	80.81	LG.7D.38	1007981	SB062	0.713	0.428	0.37			
359	WAR15	9	7D	80.81	LG.7D.38	1007981	Scout	-3.213	0.084	1.08			
360	WAR15	9	7D	80.81	LG.7D.38	1007981	SeriM82	-0.717	0.428	0.37			
361	WAR15	9	7D	80.81	LG.7D.38	1007981	Suntop	-1.785	0.217	0.66			
362	WAR15	9	7D	80.81	LG.7D.38	1007981	Westonia	-0.156	0.484	0.31			
363	WAR15	9	7D	80.81	LG.7D.38	1007981	ZWB10.37	-0.13	0.487	0.31			
364	WAR15	9	7D	80.81	LG.7D.38	1007981	ZWW10.50	0.294	0.47	0.33			
99	WAR15	4	2A	86.71	LG.2A.142	991712	Dharwah dry	0.395	0.456	0.34	0.988	0.5	0.01
100	WAR15	4	2A	86.71	LG.2A.142	991712	Drysdale	-0.27	0.47	0.33			
101	WAR15	4	2A	86.71	LG.2A.142	991712	EGA Gregory	-0.088	0.49	0.31			
102	WAR15	4	2A	86.71	LG.2A.142	991712	EGA Wylie	-0.147	0.484	0.32			
103	WAR15	4	2A	86.71	LG.2A.142	991712	FAC10.16	0.008	0.499	0.3			
104	WAR15	4	2A	86.71	LG.2A.142	991712	Mace	2.466	0.194	0.71			
105	WAR15	4	2A	86.71	LG.2A.142	991712	UQ114	0.051	0.494	0.31			
106	WAR15	4	2A	86.71	LG.2A.142	991712	SB062	0.581	0.436	0.36			
107	WAR15	4	2A	86.71	LG.2A.142	991712	Scout	0.628	0.431	0.37			
108	WAR15	4	2A	86.71	LG.2A.142	991712	SeriM82	-0.584	0.435	0.36			
109	WAR15	4	2A	86.71	LG.2A.142	991712	Suntop	0.119	0.487	0.31			
110	WAR15	4	2A	86.71	LG.2A.142	991712	Westonia	-0.127	0.486	0.31			
111	WAR15	4	2A	86.71	LG.2A.142	991712	ZWB10.37	-0.058	0.494	0.31			
112	WAR15	4	2A	86.71	LG.2A.142	991712	ZWW10.50	-2.974	0.149	0.83			
127	WAR15	7	2B	75.13	LG.2B.157	1036795	Dharwah dry	0.677	0.442	0.36	0.894	1.8	0.05
128	WAR15	7	2B	75.13	LG.2B.157	1036795	Drysdale	-0.462	0.46	0.34			
129	WAR15	7	2B	75.13	LG.2B.157	1036795	EGA Gregory	-0.15	0.487	0.31			
130	WAR15	7	2B	75.13	LG.2B.157	1036795	EGA Wylie	-4.029	0.134	0.87			
131	WAR15	7	2B	75.13	LG.2B.157	1036795	FAC10.16	0.014	0.499	0.3			
132	WAR15	7	2B	75.13	LG.2B.157	1036795	Mace	-1.28	0.39	0.41			
133	WAR15	7	2B	75.13	LG.2B.157	1036795	UQ114	0.087	0.493	0.31			
134	WAR15	7	2B	75.13	LG.2B.157	1036795	SB062	0.995	0.415	0.38			
135	WAR15	7	2B	75.13	LG.2B.157	1036795	Scout	1.076	0.408	0.39			
136	WAR15	7	2B	75.13	LG.2B.157	1036795	SeriM82	-1.001	0.414	0.38			
137	WAR15	7	2B	75.13	LG.2B.157	1036795	Suntop	3.981	0.135	0.87			
138	WAR15	7	2B	75.13	LG.2B.157	1036795	Westonia	-0.218	0.481	0.32			
139	WAR15	7	2B	75.13	LG.2B.157	1036795	ZWB10.37	-0.1	0.491	0.31			
140	WAR15	7	2B	75.13	LG.2B.157	1036795	ZWW10.50	0.41	0.464	0.33			
141	WAR15	16	2B	81.52	LG.2B.117	1091982	Dharwah dry	0.074	0.481	0.32	1	0.1	0
142	WAR15	16	2B	81.52	LG.2B.117	1091982	Drysdale	1.342	0.141	0.85			
143	WAR15	16	2B	81.52	LG.2B.117	1091982	EGA Gregory	-0.016	0.496	0.3			
144	WAR15	16	2B	81.52	LG.2B.117	1091982	EGA Wylie	-0.028	0.493	0.31			
145	WAR15	16	2B	81.52	LG.2B.117	1091982	FAC10.16	0.002	0.5	0.3			
146	WAR15	16	2B	81.52	LG.2B.117	1091982	Mace	-0.371	0.398	0.4			
147	WAR15	16	2B	81.52	LG.2B.117	1091982	UQ114	0.009	0.498	0.3			
148	WAR15	16	2B	81.52	LG.2B.117	1091982	SB062	0.109	0.472	0.33			
149	WAR15	16	2B	81.52	LG.2B.117	1091982	Scout	-0.617	0.335	0.48			
150	WAR15	16	2B	81.52	LG.2B.117	1091982	SeriM82	-0.109	0.472	0.33			
151	WAR15	16	2B	81.52	LG.2B.117	1091982	Suntop	-0.404	0.386	0.41			
152	WAR15	16	2B	81.52	LG.2B.117	1091982	Westonia	-0.024	0.494	0.31			
153	WAR15	16	2B	81.52	LG.2B.117	1091982	ZWB10.37	-0.011	0.497	0.3			
154	WAR15	16	2B	81.52	LG.2B.117	1091982	ZWW10.50	0.045	0.489	0.31			
169	WAR15	11	3A	47.73	LG.3A.69	1096770	Dharwah dry	0.154	0.473	0.33	1	0.2	0
170	WAR15	11	3A	47.73	LG.3A.69	1096770	Drysdale	-0.105	0.481	0.32			
171	WAR15	11	3A	47.73	LG.3A.69	1096770	EGA Gregory	-0.034	0.494	0.31			
172	WAR15	11	3A	47.73	LG.3A.69	1096770	EGA Wylie	-1.327	0.248	0.61			
173	WAR15	11	3A	47.73	LG.3A.69	1096770	FAC10.16	0.003	0.499	0.3			
174	WAR15	11	3A	47.73	LG.3A.69	1096770	Mace	-0.292	0.449	0.35			
175	WAR15	11	3A	47.73	LG.3A.69	1096770	UQ114	0.02	0.497	0.3			
176	WAR15	11	3A	47.73	LG.3A.69	1096770	SB062	0.227	0.46	0.34			
177	WAR15	11	3A	47.73	LG.3A.69	1096770	Scout	0.245	0.457	0.34			
178	WAR15	11	3A	47.73	LG.3A.69	1096770	SeriM82	-0.228	0.46	0.34			
179	WAR15	11	3A	47.73	LG.3A.69	1096770	Suntop	1.316	0.25	0.6			
180	WAR15	11	3A	47.73	LG.3A.69	1096770	Westonia	-0.05	0.491	0.31			
181	WAR15	11	3A	47.73	LG.3A.69	1096770	ZWB10.37	-0.023	0.496	0.3			
182	WAR15	11	3A	47.73	LG.3A.69	1096770	ZWW10.50	0.093	0.484	0.32			
197	WAR15	25	3B	111.3	LG.3B.164	1167676	Dharwah dry	2.396	0.054	1.27	0.933	0.4	0.03
198	WAR15	25	3B	111.3	LG.3B.164	1167676	Drysdale	-0.105	0.481	0.32			
199	WAR15	25	3B	111.3	LG.3B.164	1167676	EGA Gregory	-0.034	0.494	0.31			
200	WAR15	25	3B	111.3	LG.3B.164	1167676	EGA Wylie	-0.057	0.49	0.31			

								Founder Specific				Overall	
Order	Experiment	Iteration	LG	Distance (cM)	Marker	CloneID	Founder	Size	Probability	Score	Probability2	% var	Score2
201	WAR15	25	3B	111.3	LG.3B.164	1167676	FAC10.16	0.003	0.499	0.3			
202	WAR15	25	3B	111.3	LG.3B.164	1167676	Mace	-1.632	0.193	0.71			
203	WAR15	25	3B	111.3	LG.3B.164	1167676	UQ114	0.02	0.497	0.3			
204	WAR15	25	3B	111.3	LG.3B.164	1167676	SB062	0.226	0.46	0.34			
205	WAR15	25	3B	111.3	LG.3B.164	1167676	Scout	0.282	0.437	0.36			
206	WAR15	25	3B	111.3	LG.3B.164	1167676	SeriM82	-0.227	0.46	0.34			
207	WAR15	25	3B	111.3	LG.3B.164	1167676	Suntop	-0.893	0.309	0.51			
208	WAR15	25	3B	111.3	LG.3B.164	1167676	Westonia	-0.05	0.491	0.31			
209	WAR15	25	3B	111.3	LG.3B.164	1167676	ZWB10.37	-0.023	0.496	0.3			
210	WAR15	25	3B	111.3	LG.3B.164	1167676	ZWW10.50	0.093	0.484	0.32			
239	WAR15	8	5A	67.87	LG.5A.143	3022342	Dharwah dry	-0.072	0.48	0.32	0.972	0.2	0.01
240	WAR15	8	5A	67.87	LG.5A.143	3022342	Drysdale	-0.056	0.484	0.31			
241	WAR15	8	5A	67.87	LG.5A.143	3022342	EGA Gregory	-0.017	0.496	0.3			
242	WAR15	8	5A	67.87	LG.5A.143	3022342	EGA Wylie	-0.029	0.493	0.31			
243	WAR15	8	5A	67.87	LG.5A.143	3022342	FAC10.16	0.002	0.5	0.3			
244	WAR15	8	5A	67.87	LG.5A.143	3022342	Mace	-1.636	0.057	1.24			
245	WAR15	8	5A	67.87	LG.5A.143	3022342	UQ114	0.01	0.498	0.3			
246	WAR15	8	5A	67.87	LG.5A.143	3022342	SB062	1.28	0.18	0.74			
247	WAR15	8	5A	67.87	LG.5A.143	3022342	Scout	0.125	0.469	0.33			
248	WAR15	8	5A	67.87	LG.5A.143	3022342	SeriM82	0.737	0.3	0.52			
249	WAR15	8	5A	67.87	LG.5A.143	3022342	Suntop	0.024	0.494	0.31			
250	WAR15	8	5A	67.87	LG.5A.143	3022342	Westonia	-0.658	0.325	0.49			
251	WAR15	8	5A	67.87	LG.5A.143	3022342	ZWB10.37	-0.012	0.497	0.3			
252	WAR15	8	5A	67.87	LG.5A.143	3022342	ZWW10.50	0.303	0.419	0.38			
309	WAR15	3	7A	88.85	LG.7A.110	1180442	Dharwah dry	0.168	0.472	0.33	0.903	0.6	0.04
310	WAR15	3	7A	88.85	LG.7A.110	1180442	Drysdale	2.437	0.059	1.23			
311	WAR15	3	7A	88.85	LG.7A.110	1180442	EGA Gregory	-0.037	0.494	0.31			
312	WAR15	3	7A	88.85	LG.7A.110	1180442	EGA Wylie	-0.063	0.489	0.31			
313	WAR15	3	7A	88.85	LG.7A.110	1180442	FAC10.16	0.004	0.499	0.3			
314	WAR15	3	7A	88.85	LG.7A.110	1180442	Mace	-0.191	0.459	0.34			
315	WAR15	3	7A	88.85	LG.7A.110	1180442	UQ114	0.022	0.496	0.3			
316	WAR15	3	7A	88.85	LG.7A.110	1180442	SB062	0.247	0.458	0.34			
317	WAR15	3	7A	88.85	LG.7A.110	1180442	Scout	-0.341	0.431	0.37			
318	WAR15	3	7A	88.85	LG.7A.110	1180442	SeriM82	-0.248	0.458	0.34			
319	WAR15	3	7A	88.85	LG.7A.110	1180442	Suntop	-2.02	0.138	0.86			
320	WAR15	3	7A	88.85	LG.7A.110	1180442	Westonia	-0.054	0.491	0.31			
321	WAR15	3	7A	88.85	LG.7A.110	1180442	ZWB10.37	-0.025	0.496	0.3			
322	WAR15	3	7A	88.85	LG.7A.110	1180442	ZWW10.50	0.102	0.483	0.32			
323	WAR15	6	7A	89.5	LG.7A.127	1204762	Dharwah dry	0.682	0.441	0.36	0.783	1.1	0.11
324	WAR15	6	7A	89.5	LG.7A.127	1204762	Drysdale	-0.465	0.459	0.34			
325	WAR15	6	7A	89.5	LG.7A.127	1204762	EGA Gregory	-0.151	0.487	0.31			
326	WAR15	6	7A	89.5	LG.7A.127	1204762	EGA Wylie	-0.254	0.478	0.32			
327	WAR15	6	7A	89.5	LG.7A.127	1204762	FAC10.16	0.014	0.499	0.3			
328	WAR15	6	7A	89.5	LG.7A.127	1204762	Mace	3.684	0.149	0.83			
329	WAR15	6	7A	89.5	LG.7A.127	1204762	UQ114	0.087	0.493	0.31			
330	WAR15	6	7A	89.5	LG.7A.127	1204762	SB062	1.002	0.414	0.38			
331	WAR15	6	7A	89.5	LG.7A.127	1204762	Scout	1.083	0.408	0.39			
332	WAR15	6	7A	89.5	LG.7A.127	1204762	SeriM82	-1.008	0.413	0.38			
333	WAR15	6	7A	89.5	LG.7A.127	1204762	Suntop	0.205	0.482	0.32			
334	WAR15	6	7A	89.5	LG.7A.127	1204762	Westonia	-0.22	0.481	0.32			
335	WAR15	6	7A	89.5	LG.7A.127	1204762	ZWB10.37	-0.101	0.491	0.31			
336	WAR15	6	7A	89.5	LG.7A.127	1204762	ZWW10.50	-4.56	0.101	0.99			
1	WAR16	2	2B	63.52	LG.2B.98	4989040	Dharwah dry	1.515	0.358	0.45	0.069	11.1	1.16
2	WAR16	2	2B	63.52	LG.2B.98	4989040	Drysdale	-1.571	0.274	0.56			
3	WAR16	2	2B	63.52	LG.2B.98	4989040	EGA Gregory	-0.226	0.479	0.32			
4	WAR16	2	2B	63.52	LG.2B.98	4989040	EGA Wylie	-1.943	0.276	0.56			
5	WAR16	2	2B	63.52	LG.2B.98	4989040	FAC10.16	0.102	0.491	0.31			
6	WAR16	2	2B	63.52	LG.2B.98	4989040	Mace	-2.723	0.175	0.76			
7	WAR16	2	2B	63.52	LG.2B.98	4989040	UQ114	-0.62	0.442	0.35			
8	WAR16	2	2B	63.52	LG.2B.98	4989040	SB062	0.345	0.467	0.33			
9	WAR16	2	2B	63.52	LG.2B.98	4989040	SeriM82	-0.988	0.407	0.39			
10	WAR16	2	2B	63.52	LG.2B.98	4989040	Suntop	6.796	0.005	2.28			
11	WAR16	2	2B	63.52	LG.2B.98	4989040	Westonia	-0.438	0.459	0.34			
12	WAR16	2	2B	63.52	LG.2B.98	4989040	ZWB10.37	-0.25	0.477	0.32			
13	WAR16	2	2B	63.52	LG.2B.98	4989040	ZWW10.50	0	0.5	0.3			
14	WAR16	5	2B	107.01	LG.2B.408	1218896	Dharwah dry	-2.168	0.174	0.76	0.049	3.6	1.31
15	WAR16	5	2B	107.01	LG.2B.408	1218896	Drysdale	-1.895	0.197	0.71			
16	WAR16	5	2B	107.01	LG.2B.408	1218896	EGA Gregory	1.685	0.261	0.58			
17	WAR16	5	2B	107.01	LG.2B.408	1218896	EGA Wylie	-0.184	0.478	0.32			
18	WAR16	5	2B	107.01	LG.2B.408	1218896	FAC10.16	0.06	0.493	0.31			
19	WAR16	5	2B	107.01	LG.2B.408	1218896	Mace	2.653	0.056	1.25			
20	WAR16	5	2B	107.01	LG.2B.408	1218896	UQ114	-0.366	0.457	0.34			
21	WAR16	5	2B	107.01	LG.2B.408	1218896	SB062	3.924	0.043	1.37			
22	WAR16	5	2B	107.01	LG.2B.408	1218896	SeriM82	-4.3	0.028	1.55			
23	WAR16	5	2B	107.01	LG.2B.408	1218896	Suntop	-0.474	0.428	0.37			
24	WAR16	5	2B	107.01	LG.2B.408	1218896	Westonia	1.211	0.314	0.5			
25	WAR16	5	2B	107.01	LG.2B.408	1218896	ZWB10.37	-0.147	0.482	0.32			
26	WAR16	5	2B	107.01	LG.2B.408	1218896	ZWW10.50	0	0.5	0.3			
27	WAR16	3	3A	151.45	LG.3A.193	1021077	Dharwah dry	0.849	0.396	0.4	0.006	3	2.23
28	WAR16	3	3A	151.45	LG.3A.193	1021077	Drysdale	5.129	0.003	2.54			
29	WAR16	3	3A	151.45	LG.3A.193	1021077	EGA Gregory	-0.127	0.485	0.31			

								Founder Specific			Overall		
Order	Experiment	Iteration	LG	Distance (cM)	Marker	CloneID	Founder	Size	Probability	Score	Probability2	% var	Score2
30	WAR16	3	3A	151.45	LG.3A.193	1021077	EGA Wylie	-3.662	0.069	1.16			
31	WAR16	3	3A	151.45	LG.3A.193	1021077	FAC10.16	0.057	0.493	0.31			
32	WAR16	3	3A	151.45	LG.3A.193	1021077	Mace	-1.888	0.134	0.87			
33	WAR16	3	3A	151.45	LG.3A.193	1021077	UQ114	-0.348	0.458	0.34			
34	WAR16	3	3A	151.45	LG.3A.193	1021077	SB062	2.737	0.071	1.15			
35	WAR16	3	3A	151.45	LG.3A.193	1021077	SeriM82	0.124	0.474	0.32			
36	WAR16	3	3A	151.45	LG.3A.193	1021077	Suntop	-1.026	0.265	0.58			
37	WAR16	3	3A	151.45	LG.3A.193	1021077	Westonia	-1.336	0.294	0.53			
38	WAR16	3	3A	151.45	LG.3A.193	1021077	ZWB10.37	-0.51	0.434	0.36			
39	WAR16	3	3A	151.45	LG.3A.193	1021077	ZWW10.50	0	0.5	0.3			
40	WAR16	10	3D	149.01	LG.3D.42	998291	Dharwah dry	3.012	0.073	1.14	0.826	1.5	0.08
41	WAR16	10	3D	149.01	LG.3D.42	998291	Drysdale	0.403	0.446	0.35			
42	WAR16	10	3D	149.01	LG.3D.42	998291	EGA Gregory	-0.105	0.486	0.31			
43	WAR16	10	3D	149.01	LG.3D.42	998291	EGA Wylie	-0.144	0.481	0.32			
44	WAR16	10	3D	149.01	LG.3D.42	998291	FAC10.16	0.047	0.494	0.31			
45	WAR16	10	3D	149.01	LG.3D.42	998291	Mace	-2.689	0.121	0.92			
46	WAR16	10	3D	149.01	LG.3D.42	998291	UQ114	-0.287	0.462	0.34			
47	WAR16	10	3D	149.01	LG.3D.42	998291	SB062	0.16	0.479	0.32			
48	WAR16	10	3D	149.01	LG.3D.42	998291	SeriM82	-0.457	0.439	0.36			
49	WAR16	10	3D	149.01	LG.3D.42	998291	Suntop	0.379	0.435	0.36			
50	WAR16	10	3D	149.01	LG.3D.42	998291	Westonia	-0.203	0.473	0.33			
51	WAR16	10	3D	149.01	LG.3D.42	998291	ZWB10.37	-0.115	0.485	0.31			
52	WAR16	10	3D	149.01	LG.3D.42	998291	ZWW10.50	0	0.5	0.3			
53	WAR16	7	4B	35.55	LG.4B.34	4010028	Dharwah dry	-1.682	0.247	0.61	0.172	3.9	0.76
54	WAR16	7	4B	35.55	LG.4B.34	4010028	Drysdale	3.564	0.065	1.18			
55	WAR16	7	4B	35.55	LG.4B.34	4010028	EGA Gregory	-0.122	0.485	0.31			
56	WAR16	7	4B	35.55	LG.4B.34	4010028	EGA Wylie	-0.168	0.479	0.32			
57	WAR16	7	4B	35.55	LG.4B.34	4010028	FAC10.16	0.055	0.493	0.31			
58	WAR16	7	4B	35.55	LG.4B.34	4010028	Mace	3.339	0.065	1.19			
59	WAR16	7	4B	35.55	LG.4B.34	4010028	UQ114	-0.335	0.459	0.34			
60	WAR16	7	4B	35.55	LG.4B.34	4010028	SB062	-1.883	0.219	0.66			
61	WAR16	7	4B	35.55	LG.4B.34	4010028	SeriM82	-0.079	0.487	0.31			
62	WAR16	7	4B	35.55	LG.4B.34	4010028	Suntop	-2.319	0.138	0.86			
63	WAR16	7	4B	35.55	LG.4B.34	4010028	Westonia	-0.236	0.471	0.33			
64	WAR16	7	4B	35.55	LG.4B.34	4010028	ZWB10.37	-0.135	0.483	0.32			
65	WAR16	7	4B	35.55	LG.4B.34	4010028	ZWW10.50	0	0.5	0.3			
66	WAR16	6	4D	50.96	LG.4D.16	1161775	Dharwah dry	1.803	0.219	0.66	0.118	3.7	0.93
67	WAR16	6	4D	50.96	LG.4D.16	1161775	Drysdale	-1.002	0.326	0.49			
68	WAR16	6	4D	50.96	LG.4D.16	1161775	EGA Gregory	-0.133	0.484	0.32			
69	WAR16	6	4D	50.96	LG.4D.16	1161775	EGA Wylie	-0.183	0.478	0.32			
70	WAR16	6	4D	50.96	LG.4D.16	1161775	FAC10.16	0.06	0.493	0.31			
71	WAR16	6	4D	50.96	LG.4D.16	1161775	Mace	-3.025	0.035	1.46			
72	WAR16	6	4D	50.96	LG.4D.16	1161775	UQ114	-0.366	0.457	0.34			
73	WAR16	6	4D	50.96	LG.4D.16	1161775	SB062	-2.085	0.182	0.74			
74	WAR16	6	4D	50.96	LG.4D.16	1161775	SeriM82	4.911	0.016	1.78			
75	WAR16	6	4D	50.96	LG.4D.16	1161775	Suntop	1.344	0.343	0.47			
76	WAR16	6	4D	50.96	LG.4D.16	1161775	Westonia	-1.176	0.317	0.5			
77	WAR16	6	4D	50.96	LG.4D.16	1161775	ZWB10.37	-0.147	0.482	0.32			
78	WAR16	6	4D	50.96	LG.4D.16	1161775	ZWW10.50	0	0.5	0.3			
79	WAR16	11	5B	30.46	LG.5B.89	1092379	Dharwah dry	-0.369	0.399	0.4	0.696	1	0.16
80	WAR16	11	5B	30.46	LG.5B.89	1092379	Drysdale	-0.348	0.401	0.4			
81	WAR16	11	5B	30.46	LG.5B.89	1092379	EGA Gregory	-0.03	0.493	0.31			
82	WAR16	11	5B	30.46	LG.5B.89	1092379	EGA Wylie	-0.13	0.464	0.33			
83	WAR16	11	5B	30.46	LG.5B.89	1092379	FAC10.16	0.156	0.455	0.34			
84	WAR16	11	5B	30.46	LG.5B.89	1092379	Mace	0.269	0.426	0.37			
85	WAR16	11	5B	30.46	LG.5B.89	1092379	UQ114	0.317	0.411	0.39			
86	WAR16	11	5B	30.46	LG.5B.89	1092379	SB062	-0.08	0.477	0.32			
87	WAR16	11	5B	30.46	LG.5B.89	1092379	SeriM82	-1.031	0.232	0.63			
88	WAR16	11	5B	30.46	LG.5B.89	1092379	Suntop	2.254	0.01	2			
89	WAR16	11	5B	30.46	LG.5B.89	1092379	Westonia	-0.057	0.486	0.31			
90	WAR16	11	5B	30.46	LG.5B.89	1092379	ZWB10.37	-0.95	0.252	0.6			
91	WAR16	11	5B	30.46	LG.5B.89	1092379	ZWW10.50	0	0.5	0.3			
92	WAR16	9	6A	17.57	LG.6A.23	1096778	Dharwah dry	-3.2	0.119	0.92	0.497	4.7	0.3
93	WAR16	9	6A	17.57	LG.6A.23	1096778	Drysdale	0.766	0.423	0.37			
94	WAR16	9	6A	17.57	LG.6A.23	1096778	EGA Gregory	-0.199	0.48	0.32			
95	WAR16	9	6A	17.57	LG.6A.23	1096778	EGA Wylie	-0.274	0.473	0.33			
96	WAR16	9	6A	17.57	LG.6A.23	1096778	FAC10.16	0.089	0.491	0.31			
97	WAR16	9	6A	17.57	LG.6A.23	1096778	Mace	5.432	0.034	1.47			
98	WAR16	9	6A	17.57	LG.6A.23	1096778	UQ114	-0.546	0.446	0.35			
99	WAR16	9	6A	17.57	LG.6A.23	1096778	SB062	0.304	0.47	0.33			
100	WAR16	9	6A	17.57	LG.6A.23	1096778	SeriM82	-0.869	0.414	0.38			
101	WAR16	9	6A	17.57	LG.6A.23	1096778	Suntop	-0.898	0.381	0.42			
102	WAR16	9	6A	17.57	LG.6A.23	1096778	Westonia	-0.386	0.462	0.34			
103	WAR16	9	6A	17.57	LG.6A.23	1096778	ZWB10.37	-0.22	0.478	0.32			
104	WAR16	9	6A	17.57	LG.6A.23	1096778	ZWW10.50	0	0.5	0.3			
105	WAR16	1	6B	78.29	LG.6B.204	4539744	Dharwah dry	2.481	0.313	0.5	0.002	17.6	2.74
106	WAR16	1	6B	78.29	LG.6B.204	4539744	Drysdale	4.418	0.093	1.03			
107	WAR16	1	6B	78.29	LG.6B.204	4539744	EGA Gregory	-0.37	0.472	0.33			
108	WAR16	1	6B	78.29	LG.6B.204	4539744	EGA Wylie	-0.509	0.461	0.34			
109	WAR16	1	6B	78.29	LG.6B.204	4539744	FAC10.16	0.166	0.487	0.31			
110	WAR16	1	6B	78.29	LG.6B.204	4539744	Mace	4.69	0.095	1.02			

								Founder Specific			Overall		
Order	Experiment	Iteration	LG	Distance (cM)	Marker	CloneID	Founder	Size	Probability	Score	Probability2	% var	Score2
111	WAR16	1	6B	78.29	LG.6B.204	4539744	UQ114	-1.016	0.424	0.37			
112	WAR16	1	6B	78.29	LG.6B.204	4539744	SB062	0.566	0.456	0.34			
113	WAR16	1	6B	78.29	LG.6B.204	4539744	SeriM82	-1.618	0.377	0.42			
114	WAR16	1	6B	78.29	LG.6B.204	4539744	Suntop	-7.682	0.016	1.81			
115	WAR16	1	6B	78.29	LG.6B.204	4539744	Westonia	-0.717	0.445	0.35			
116	WAR16	1	6B	78.29	LG.6B.204	4539744	ZWB10.37	-0.409	0.469	0.33			
117	WAR16	1	6B	78.29	LG.6B.204	4539744	ZWW10.50	0	0.5	0.3			
118	WAR16	4	7A	149.56	LG.7A.216	1216524	Dharwah dry	5.686	0.03	1.53	0.008	9.5	2.11
119	WAR16	4	7A	149.56	LG.7A.216	1216524	Drysdale	-6.715	0.009	2.05			
120	WAR16	4	7A	149.56	LG.7A.216	1216524	EGA Gregory	-0.292	0.476	0.32			
121	WAR16	4	7A	149.56	LG.7A.216	1216524	EGA Wylie	-0.402	0.466	0.33			
122	WAR16	4	7A	149.56	LG.7A.216	1216524	FAC10.16	0.131	0.489	0.31			
123	WAR16	4	7A	149.56	LG.7A.216	1216524	Mace	-3.424	0.064	1.19			
124	WAR16	4	7A	149.56	LG.7A.216	1216524	UQ114	-0.801	0.434	0.36			
125	WAR16	4	7A	149.56	LG.7A.216	1216524	SB062	-1.378	0.318	0.5			
126	WAR16	4	7A	149.56	LG.7A.216	1216524	SeriM82	5.032	0.044	1.36			
127	WAR16	4	7A	149.56	LG.7A.216	1216524	Suntop	2.945	0.264	0.58			
128	WAR16	4	7A	149.56	LG.7A.216	1216524	Westonia	-0.459	0.44	0.36			
129	WAR16	4	7A	149.56	LG.7A.216	1216524	ZWB10.37	-0.322	0.473	0.33			
130	WAR16	4	7A	149.56	LG.7A.216	1216524	ZWW10.50	0	0.5	0.3			
131	WAR16	8	7B	104.5	LG.7B.176	1089670	Dharwah dry	5.243	0.041	1.39	0.162	7.1	0.79
132	WAR16	8	7B	104.5	LG.7B.176	1089670	Drysdale	1.041	0.408	0.39			
133	WAR16	8	7B	104.5	LG.7B.176	1089670	EGA Gregory	-0.27	0.477	0.32			
134	WAR16	8	7B	104.5	LG.7B.176	1089670	EGA Wylie	-0.372	0.468	0.33			
135	WAR16	8	7B	104.5	LG.7B.176	1089670	FAC10.16	0.122	0.49	0.31			
136	WAR16	8	7B	104.5	LG.7B.176	1089670	Mace	-5.618	0.048	1.31			
137	WAR16	8	7B	104.5	LG.7B.176	1089670	UQ114	-0.742	0.436	0.36			
138	WAR16	8	7B	104.5	LG.7B.176	1089670	SB062	0.413	0.464	0.33			
139	WAR16	8	7B	104.5	LG.7B.176	1089670	SeriM82	-1.181	0.398	0.4			
140	WAR16	8	7B	104.5	LG.7B.176	1089670	Suntop	2.187	0.253	0.6			
141	WAR16	8	7B	104.5	LG.7B.176	1089670	Westonia	-0.524	0.454	0.34			
142	WAR16	8	7B	104.5	LG.7B.176	1089670	ZWB10.37	-0.298	0.474	0.32			
143	WAR16	8	7B	104.5	LG.7B.176	1089670	ZWW10.50	0	0.5	0.3			
